# Influence of ATP-Binding Cassette Transporter 1 R219K and M883I Polymorphisms on Development of Atherosclerosis: A Meta-Analysis of 58 Studies

**DOI:** 10.1371/journal.pone.0086480

**Published:** 2014-01-23

**Authors:** Yan-Wei Yin, Jing-Cheng Li, Dong Gao, Yan-Xiu Chen, Bing-Hu Li, Jing-Zhou Wang, Yun Liu, Shao-Qiong Liao, Ming-Jie Zhang, Chang-Yue Gao, Li-Li Zhang

**Affiliations:** 1 Department of Neurology, Institute of Surgery Research, Daping Hospital, Third Military Medical University, Yuzhong District, Chongqing, PR China; 2 Department of Neurology, The brain hospital of Liaocheng Hospital, Liaocheng, Shandong, PR China; Nanjing Medical University, China

## Abstract

**Background:**

Numerous epidemiological studies have evaluated the associations between ATP-binding cassette transporter 1 (ABCA1) R219K (rs2230806) and M883I (rs4149313) polymorphisms and atherosclerosis (AS), but results remain controversial. The purpose of the present study is to investigate whether these two polymorphisms facilitate the susceptibility to AS using a meta-analysis.

**Methods:**

PubMed, Embase, Web of Science, Medline, Cochrane database, Clinicaltrials.gov, Current Controlled Trials, Chinese Clinical Trial Registry, CBMdisc, CNKI, Google Scholar and Baidu Library were searched to get the genetic association studies. All statistical analyses were done with Stata 11.0.

**Results:**

Forty-seven articles involving 58 studies were included in the final meta-analysis. For the ABCA1 R219K polymorphism, 42 studies involving 12,551 AS cases and 19,548 controls were combined showing significant association between this variant and AS risk (for K allele vs. R allele: OR = 0.77, 95% CI = 0.71–0.84, *P*<0.01; for K/K vs. R/R: OR = 0.60, 95% CI = 0.51–0.71, *P*<0.01; for K/K vs. R/K+R/R: OR = 0.69, 95% CI = 0.60–0.80, *P*<0.01; for K/K+R/K vs. R/R: OR = 0.74, 95% CI = 0.66–0.83, *P*<0.01). For the ABCA1 M883I polymorphism, 16 studies involving 4,224 AS cases and 3,462 controls were combined. There was also significant association between the variant and AS risk (for I allele vs. M allele: OR = 0.85, 95% CI = 0.77–0.95, *P*<0.01).

**Conclusions:**

The present meta-analysis suggested that the ABCA1 R219K and M883I polymorphisms were associated with the susceptibility to AS. However, due to the high heterogeneity in the meta-analysis, the results should be interpreted with caution.

## Introduction

Atherosclerosis (AS) is a common, multifactorial disease that causes significant morbidity and mortality worldwide [1]. Its exact mechanisms are still unclear. Multiple environmental and lifestyle factors may significantly contribute to the AS risk, such as climate, diet, age, and economic status. The risk of developing AS tends to run in families, which suggests the genetic factors play a crucial role in the development of AS. Until recently, many studies have focused on this field, and ATP-binding cassette transporter 1 (ABCA1) gene has been extensively studied [2]–[48].

It is widely accepted that a low level of plasma high-density lipoprotein cholesterol (HDL-C) is a major risk factor for atherosclerotic diseases in the general population [49]. ABCA1, a member of the ATP-binding cassette family,could promote the HDL formation and the cholesterol efflux, thus impacting the development of AS [50]. Previous studies have reported that the mutations in ABCA1 are responsible for two forms of heritable HDL disorders, namely Tangier disease (Homozygosity) and familial hypoalphalipoproteinemia (heterozygosity) [51], [52]. Given the crucial role of ABCA1 in the HDL formation and the cholesterol efflux, the mutations in ABCA1 may also play a significant role in the development of atherosclerotic diseases. Recently, a number of molecular epidemiological studies have been done to evaluate the associations between the ABCA1 gene polymorphisms (such as R219K, M883I, C69T, V825I, R1587K, V771M and −565C/T) and the risk of atherosclerotic diseases [2]–[48]. However, results of different studies have been inconsistent, possibly due to small sample sizes in the individual studies. In 2011, Ma et al. performed a meta-analysis to evaluate the association between the ABCA1 R219K polymorphism and coronary artery disease (CAD), and demonstrated that the ABCA1 R219K polymorphism was a protective role for CAD both in Asians and Caucasians [53]. In 2012, Li et al. also conducted a meta-analysis to evaluate the association of this variant with CAD [54]. However, they just found the ABCA1 R219K polymorphism was a protective factor in Asians, but not in Caucasians. Considering the above two meta-analyses only focused on the association of ABCA1 R219K polymorphism with the single atherosclerotic disease, we therefore performed this meta-analysis of all the studies available now to derive a more precise estimation of the associations between the ABCA1 R219K and M883I polymorphisms and overall AS risk.

## Materials and Methods

### Literature Search

This meta-analysis followed the Preferred Reporting Items for Systematic Reviews and Meta-analyses (PRISMA) criteria and Meta-analysis of Observational Studies in Epidemiology (MOOSE) guidelines [55], [56]. Eligible literatures published before the end of November 15, 2013 were identified by the search of PubMed, Embase, Web of Science, Medline, Cochrane database, Clinicaltrials.gov, Current Controlled Trials, Chinese Clinical Trial Registry, CBMdisc, CNKI, Google Scholar and Baidu Library using combinations of the following keywords: (“ATP-binding cassette transporter A1” OR “ATP-binding cassette sub-family A member-1” OR “ABCA1”) AND (“polymorphism” OR “mutation” OR “variant” OR “variation” OR “genotype”) AND (“coronary artery disease” OR “CAD” OR “coronary heart disease” OR “CHD” OR “myocardial infarction” OR “MI” OR “ischemic cardiovascular disease” OR “ischemic cardiovascular events” OR “ischemic stroke” OR “IS” OR “cerebrovascular disease” OR “ischemic cerebrovascular events” OR “cerebral infarction” OR “cerebral ischemia” OR “brain infarction” OR “carotid artery stenosis” OR “CAAD” OR “transient ischemic attack” OR “TIA” OR “peripheral Arterial Disease” OR “PAD” OR “peripheral artery occlusive disease” OR “PAOD” OR “renal artery stenosis” OR “RAS” OR “retinal artery occlusion” OR “RAO” OR “aortic aneurysm” OR “atherosclerosis”). In addition, all references cited were reviewed to identify additional studies. Two reviewers (Yin YW and Zhang LL) searched the above databases independently. Decisions were compared and disagreements about study selection were resolved by involving a third reviewer (Li JC). The search was limited to English and Chinese language papers. There was no restriction on time period, sample size, or population.

### Inclusion and Exclusion Criteria

To be included in the present meta-analysis, the studies had to comply with the following major criteria: (1) case-control or cohort studies evaluating the associations between the ABCA1 R219K and M883I polymorphisms and AS risk; (2) published studies with full text articles; (3) Diagnosises of atherosclerotic diseases were made according to the internationally recognized diagnostic criterion. The diagnosis of ischemic heart disease (CAD and MI) is accorded with the result of coronary angiography, criteria of World Health Organization (WHO), criteria of European Society of Cardiology (ESC), or criteria of American College of Cardiology/American Heart Association (ACC/AHA), and the diagnosis of IS is accorded with result of computed tomography (CT) or magnetic resonance imaging (MRI); (4) sufficient published data for calculating odds ratios (ORs) with their 95% confidence intervals (CIs).

Studies were excluded if they were: (1) Review; (2) Not conducted in humans; (3) Duplicate studies.

### Data Extraction

Data, including name of the first author, year of publication, study population (country, ethnicity), study type (case-control and cohort study), type of atherosclerotic disease, source of controls (population-based studies and hospital-based studies), sample size (total numbers of cases and controls), and number of genotypes in cases and controls, were extracted from each study by two reviewers independently (Yin YW and Zhang LL) according to the pre-specified inclusion criteria. Decisions were compared and disagreements about study selection were resolved by consensus or by involving a third reviewer (Li JC).

### Quality Assessment for Individual Studies

The quality of the individual studies was evaluated and scored by two reviewers independently based on the Newcastle-Ottawa Scale (NOS) [57]. Each study was assessed based on three broad perspectives: selection, comparability, and exposure, and each satisfactory answer received one point. The NOS ranges between zero (none of the quality criterion was met) up to nine stars (all the quality criteria were met), and the high-quality study was considered as the one with a score equal to or higher than seven. The third reviewer (Li BH) examined the results, and a consensus was reached.

### GRADE Quality Assessment

GRADE (Grades of Recommendation, Assessment, Development and Evaluation) approach was adopted to grade quality of evidence for each association [58]. The GRADE system included: level of evidence: (1) high quality; we are very confident that the true effect lies close to that of the estimate of the effect, (2) moderate quality; we are moderately confident in the effect estimate: The true effect is likely to be close to the estimate of the effect, but there is a possibility that it is substantially different, (3) low quality; our confidence in the effect estimate is limited: The true effect may be substantially different from the estimate of the effect and (4) very low quality; we have very little confidence in the effect estimate: The true effect is likely to be substantially different from the estimate of effect. Two reviewers (Wang JZ and Zhang MJ) assessed quality independently and solved disagreement by discussion.

### Statistical Analysis

For the controls of each study, Hardy-Weinberg equilibrium (HWE) was assessed using the chi-square test (*P*<0.05 was considered significant deviation from HWE). The strength of associations between the ABCA1 R219K and M883I polymorphisms and AS risk were assessed by ORs with 95% CIs. The pooled ORs were performed for allelic model (K allele vs. R allele for R219K; I allele vs. M allele for M883I), additive model (K/K vs. R/R for R219K; I/I vs. M/M for M883I), recessive model (K/K vs. R/K+R/R for R219K; I/I vs. M/I+M/M for M883I), and dominant model (K/K+R/K vs. R/R for R219K; I/I+M/I vs. M/M for M883I). Heterogeneity across individual studies was calculated using the Cochran’s Q statistic and the I^2^ statistic (*P*<0.10 and I^2^>50% indicated evidence of heterogeneity) [59], [60]. Meta-regression was performed subsequently to explore the heterogeneity sources. The pooled OR was estimated by the random-effects model. Subgroup analyses were performed based on ethnicity (Caucasians and Asians), atherosclerotic diseases (CAD and IS), source of controls (population-based studies and hospital-based studies) and study type (case-control and cohort study). Sensitivity analyses were performed based on HWE (studies without HWE were excluded), NOS score (studies with score ≤6 were excluded) and article type (master theses were excluded). Begg’s funnel plot and Egger’s regression test were conducted to identify possible publication bias in the current meta-analysis (*P*<0.05 was considered representative of statistically significant publication bias) [61]. In addition, fail-safe number (Nfs _0.05_) was calculated to explore the extent to which the “file drawer problem” may have affected study results. All statistical analyses were done with Stata software (Version 11; StataCorp LP, College Station, TX).

## Results

### Characteristics of the Studies

**Figure 1:**
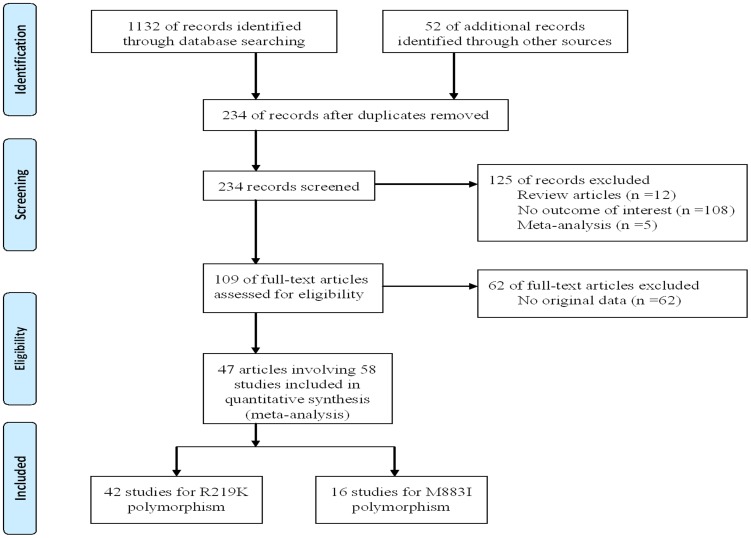
Flow diagram of the study selection process.

The present study met the PRISMA statements and MOOSE guidelines (Checklist S1, Table S1 and Fig. 1). A total of 1184 articles were identified after searching. After careful review, 47 articles involving 58 studies (42 studies for R219K polymorphism and 16 studies for M883I polymorphism) met the inclusion criteria and were selected in this meta-analysis [2]–[48]. For the ABCA1 R219K polymorphism, 12551 AS cases and 19548 controls were included to assess the association between the variant and AS risk [2]–[41]. For the ABCA1 M883I polymorphism, 4224 AS cases and 3462 controls were identified to assess the association of the variant with AS risk [14], [15], [22], [23], [29], [30], [42]–[48]. Main characteristics of the included studies were listed in Table 1 and Table 2. The most commonly atherosclerotic disease included in the present meta-analysis was CAD. In addition, there were nine studies involving IS, five studies involving MI, and one study involving IS and carotid artery atherosclerosis (CAA). There were 40 studies of Asians, 18 studies of Caucasians, and one study of mixed population. Four studies did not follow the HWE [16], [18], [42], [46] and two studies have insufficient data for calculation of the HWE [22], [29]. The NOS results showed that the average scores were 6.9 and 6.8, respectively. In addition, the results of GRADE were shown in Table S2.

**Table 1:** Characteristics of studies included in this meta-analysis.

					Study		Source of	Sample size						HWE	
Position	First author	Year	Country	Ethnicity	type	Disease	controls	(case/control)	Genotypes distribution (case/control)					Y/N(P)	Score
R219K									R/R	R/K	K/K	R	K		
	Clee	2001	Canada	Caucasians	CS	CAD	HB	432/358	248/176	170/160	14/22	666/512	198/204	Y(0.067)	6
	Brousseau	2001	USA	Caucasians	CCS	CAD	PB	1014/1013	454/543	484/402	76/68	1392/1488	636/538	Y(0.580)	7
	Cenarro	2003	Spain	Caucasians	CCS	CAD	HB	216/158	123/72	78/71	15/15	324/215	108/101	Y(0.676)	6
	Evans	2003	Germany	Caucasians	CCS	CAD	HB	114/629	72/337	35/245	7/47	179/919	49/339	Y(0.789)	7
	Harada	2003	Japan	Asian	CCS	CAD	HB	273/137	73/31	139/73	61/33	285/135	261/139	Y(0.440)	6
	Wang	2004	China	Asian	CCS	CAD	PB	222/278	79/76	94/125	49/77	252/277	192/279	Y(0.093)	7
	Zhao	2004	China	Asian	CCS	CAD	PB	236/251	96/80	111/123	29/48	303/283	169/219	Y(0.953)	8
	Wang-a	2004	China	Asian	CCS	IS	PB	58/60	23/21	27/34	8/5	73/76	43/44	Y(0.088)	7
	Wang-b	2004	China	Asian	CCS	IS	PB	58/60	19/14	35/36	4/10	73/64	43/56	Y(0.112)	7
	Xiao	2004	China	Asian	CCS	IS	PB	379/351	149/112	172/172	58/67	470/396	288/306	Y(0.947)	8
	Woll	2005	USA	Caucasians	CCS	CAD	PB	838/257	450/115	327/112	61/30	1227/342	449/172	Y(0.732)	6
	Cui	2005	China	Asian	CCS	IS	PB	96/90	15/21	35/45	46/24	65/87	127/93	Y(0.992)	7
	Whiting	2005	USA	Mixed	CS	CAD	HB	2468/834	1298/449	975/318	195/67	3571/1216	1365/452	Y(0.313)	6
	Sun	2005	China	Asian	CCS	CAD	PB	224/248	105/62	85/129	34/57	295/253	153/243	Y(0.521)	8
	Li	2005	China	Asian	CCS	CAD	PB	264/278	104/83	116/132	44/63	324/298	204/258	Y(0.449)	7
	Chang	2005	China	Asian	CCS	CAD	PB	178/83	66/17	75/22	37/44	207/56	149/110	N(0.000)	7
	Wang	2006	China	Asian	CCS	CAD	PB	396/417	158/124	174/198	64/95	490/446	302/388	Y(0.350)	
	Wang	2006	China	Asian	CCS	CAD	HB	150/139	61/47	69/53	20/39	191/147	109/131	N(0.006)	6
	Wang	2006	China	Asian	CCS	CAD	PB	234/198	108/67	105/101	21/30	321/235	147/161	Y(0.422)	8
	Cha	2006	China	Asian	CCS	CAD	PB	112/108	47/34	53/52	12/22	147/120	77/96	Y(0.795)	7
	Wu	2006	China	Asian	CCS	CAD	PB	67/20	26/2	30/7	11/11	82/11	52/29	Y(0.585)	6
	Martín	2006	Spain	Caucasians	CS	MI	HB	100/100	48/49	42/40	10/11	138/138	62/62	Y(0.516)	6
	Andrikovics-a	2006	Hungary	Caucasians	CCS	IS	PB	244/193	131/97	84/73	29/23	346/267	142/119	Y(0.116)	7
	Andrikovics-b	2006	Hungary	Caucasians	CCS	CAD	PB	150/193	84/97	55/73	11/23	223/267	77/119	Y(0.116)	7
	Yu	2008	China	Asian	CCS	MI	PB	49/72	29/24	18/35	2/13	76/83	22/61	Y(0.969)	7
	Zhang	2008	China	Asian	CCS	IS	PB	177/234	43/40	87/129	47/65	173/209	181/259	Y(0.078)	7
	Wang	2008	China	Asian	CCS	CAD	HB	111/75	30/17	54/41	27/17	114/75	108/75	Y(0.419)	6
	Zhang	2008	China	Asian	CCS	MI	PB	162/186	71/49	65/93	26/44	207/191	117/181	Y(0.992)	8
	Balcerzyk	2008	Poland	Caucasians	CCS	CAD	PB	178/180	90/91	68/78	20/11	248/260	108/100	Y(0.283)	6
	Frikke-Schmidt	2008	Denmark	Caucasians	CS	CAD	PB	1107/7858	603/4260	429/3032	75/566	1635/11552	579/4164	Y(0.405)	7
	Porchay-Baldérelli	2009	France	Caucasians	CS	CAD	HB	223/2906	106/1491	102/1183	15/232	314/4165	132/1647	Y(0.901)	6
	Li	2009	China	Asian	CCS	CAD	PB	365/246	140/92	170/116	55/38	450/300	280/192	Y(0.886)	8
	Shi	2009	China	Asian	CCS	CAD	HB	132/157	49/53	60/66	23/38	158/172	106/142	Y(0.058)	7
	Doosti	2009	Iran	Asian	CCS	CAD	HB	207/94	71/17	77/38	59/39	219/72	195/116	Y(0.161)	6
	Guo	2010	China	Asian	CCS	CAD	PB	71/83	30/29	37/38	4/16	97/96	45/70	Y(0.576)	7
	Xu	2011	China	Asian	CCS	CAD	PB	246/109	95/31	128/53	23/25	318/115	174/103	Y(0.798)	8
	Yuan	2011	China	Asian	CCS	CAD	PB	60/55	22/16	28/21	10/18	72/53	48/57	Y(0.081)	7
	Yi	2011	China	Asian	CCS	IS	PB	240/240	36/45	97/109	107/86	169/199	311/281	Y(0.319)	7
	Xue	2012	China	Asian	CCS	CAA, IS	PB	182/229	70/62	91/118	21/49	231/242	133/216	Y(0.608)	8
	Wang	2012	China	Asian	CCS	CAD	PB	141/109	63/31	63/53	15/25	189/115	93/103	Y(0.798)	7
	Li	2012	China	Asian	CCS	MI	PB	150/100	52/30	74/48	24/22	178/108	122/92	Y(0.735)	7
	Xia	2012	China	Asian	CCS	CAD	HB	227/162	96/51	107/78	24/33	299/180	155/144	Y(0.750)	6
M883I									M/M	M/I	I/I	M	I		
	Tan-a	2003	Singapore	Asian	CCS	CAD	PB	364/250	168/97	169/134	27/19	505/328	223/172	N(0.003)	7
	Tan-b	2003	Singapore	Asian	CCS	CAD	PB	100/167	26/22	45/87	29/58	97/131	103/203	Y(0.231)	7
	Tan-c	2003	Singapore	Asian	CCS	CAD	PB	152/223	6/3	20/34	126/186	32/40	272/406	Y(0.322)	7
	Li	2005	China	Asian	CCS	CAD	PB	264/278	233/230	31/48	0/0	497/508	31/48	Y(0.115)	7
	Sun	2005	China	Asian	CCS	CAD	PB	224/248	119/127	91/97	14/24	329/351	119/145	Y(0.389)	8
	Andrikovics-a	2006	Hungary	Caucasians	CCS	IS	PB	244/105	176/80	61/24	7/1	413/184	75/26	Y(0.583)	7
	Andrikovics-b	2006	Hungary	Caucasians	CCS	CAD	PB	150/105	117/80	29/24	4/1	263/184	37/26	Y(0.583)	7
	Guo	2007	China	Asian	CCS	CAD	PB	112/108	54/51	50/43	8/14	158/145	66/71	Y(0.309)	7
	Tsai	2007	China	Asian	CCS	CAD	PB	205/201	124/103	65/84	16/14	313/290	97/112	Y(0.574)	7
	Jensen	2007	USA	Caucasians	CS	CAD	PB	243/482	187/352	51/120	5/10	425/824	61/140	Y(0.951)	7
	Porchay-Baldérelli	2009	France	Caucasians	CS	CAD	HB	223/2906	165/2057	52/782	6/67	382/4896	64/916	Y(0.469)	6
	Zhang	2010	China	Asian	CCS	CAD	HB	289/171	201/94	83/73	5/4	485/261	93/81	N(0.018)	6
	Mao	2011	China	Asian	CCS	CAD	PB	357/160	140/55	150/73	67/32	430/183	284/137	Y(0.388)	6
	Liu	2011	China	Asian	CCS	IS	PB	90/100	51/68	31/28	8/4	133/164	47/36	Y(0.607)	7
									M/M	M/I+I/I					
	Martín	2006	Spain	Caucasians	CS	MI	HB	100/100	69/80	31/20				–	6
	Frikke-Schmidt	2008	Denmark	Caucasians	CS	CAD	PB	1107/7858	827/6107	280/1751				–	7

CCS: case-control study, CS: cohort study, PB: population-based, HB: hospital-based, HWE: Hardy-Weinberg equilibrium, Y: yes, N: no, CAD: coronary artery disease, CAA: carotid artery atherosclerosis, IS: ischemic stroke, MI: myocardial infarction,

**Table 2:** Meta-analyses of ABCA1 R219K and M883I polymorphisms and risk of AS in each subgroup.

		Allelic model		Additive model		Recessive model		Dominant model	
Position	SS (case/control)	OR (95% CI)	P value	OR (95% CI)	P value	OR (95% CI)	P value	OR (95%CI)	P value
Overall analysis									
R219K	12551/19548	0.77[0.71,0.84]	<0.01	0.60[0.51,0.71]	<0.01	0.69[0.60,0.80]	<0.01	0.74[0.66,0.83]	<0.01
M883I	4224/13462	0.85[0.77,0.95]	<0.01	0.79 [0.61,1.01]	0.06	0.92[0.74,1.13]	0.42	0.86[0.72,1.02]	0.08
Subgroup analysis based on ethnicity									
R219K (C)	4616/13845	0.91[0.80,1.04]	0.16	0.83[0.65,1.06]	0.14	0.87[0.72,1.05]	0.14	0.90[0.76,1.07]	0.23
R219K (A)	7935/5703	0.71[0.64,0.79]	<0.01	0.52[0.42,0.63]	<0.01	0.62[0.52,0.75]	<0.01	0.66[0.59,0.74]	<0.01
M883I (C)	2067/11556	0.94[0.78,1.12]	0.48	1.24[0.67,2.29]	0.50	1.29[0.70,2.38]	0.41	1.05[0.86,1.28]	0.66
M883I (A)	2157/1906	0.82[0.72,0.93]	<0.01	0.72[0.54,0.95]	0.02	0.88[0.70,1.09]	0.25	0.75[0.61,0.91]	<0.01
Subgroup analysis based on type of diseases									
R219K (CAD)	11117/18091	0.74[0.67,0.82]	<0.01	0.56[0.47,0.67]	<0.01	0.64[0.55,0.74]	<0.01	0.72[0.64,0.82]	<0.01
R219K (IS)	1434/1457	0.94[0.75,1.17]	0.58	0.86[0.55,1.35]	0.51	1.00[0.69,1.45]	1.00	0.82[0.65,1.02]	0.08
M883I (CAD)	3890/13257	0.82[0.74,0.90]	<0.01	0.73[0.57,0.94]	0.01	0.88[0.71,1.09]	0.24	0.81[0.68,0.97]	0.02
M883I (IS)	334/205	1.43[1.02,2.02]	0.04	2.79[0.95,8.21]	0.06	2.51[0.86,7.29]	0.09	1.40[0.94,2.07]	0.10
Subgroup analysis based on source of controls									
R219K (PB)	7898/13799	0.76[0.67,0.85]	<0.01	0.58[0.47,0.72]	<0.01	0.68[0.56,0.82]	<0.01	0.72[0.62,0.83]	<0.01
R219K (HB)	4653/5749	0.81[0.71,0.92]	<0.01	0.65[0.51,0.82]	<0.01	0.72[0.59,0.87]	<0.01	0.80[0.68,0.94]	<0.01
M883I (PB)	3612/10285	0.87[0.78,0.97]	0.01	0.77[0.58,1.04]	0.09	0.91[0.73,1.13]	0.39	0.86[0.72,1.04]	0.11
M883I (HB)	612/3177	0.75[0.52,1.08]^a^	0.13	0.93[0.45,1.90]	0.84	1.02[0.50,2.09]	0.95	0.88[0.51,1.55]	0.67
Subgroup analysis based on study type									
R219K (CS)	4330/12056	0.97[0.88,1.07]	0.55	0.91[0.75,1.10]	0.32	0.91[0.76,1.07]	0.25	0.99[0.87,1.12]	0.84
R219K (CCS)	8221/7492	0.74[0.67,0.82]	<0.01	0.57[0.47,0.68]	<0.01	0.67[0.57,0.78]	<0.01	0.70[0.62,0.79]	<0.01
M883I (CS)	1673/11346	0.87[0.71,1.08]	0.21	1.05[0.54,2.04]	0.89	1.10[0.56,2.14]	0.78	1.04[0.80,1.36]	0.77
M883I (CCS)	2551/2116	0.85[0.75,0.97]	0.01	0.75[0.56,1.02]	0.06	0.90[0.72,1.12]	0.34	0.79[0.65,0.95]	0.01
Sensitivity analysis									
R219K (BS)	7061/14128	0.76[0.68,0.85]	<0.01	0.60[0.49,0.73]	<0.01	0.69[0.58,0.82]	<0.01	0.72[0.62,0.83]	<0.01
R219K (BH)	12223/19326	0.79[0.72,0.86]	<0.01	0.62[0.53,0.74]	<0.01	0.73[0.64,0.83]	<0.01	0.75[0.67,0.84]	<0.01
R219K (BT)	11647/18669	0.78[0.71,0.85]	<0.01	0.60[0.51,0.71]	<0.01	0.70[0.61,0.80]	<0.01	0.74[0.66,0.83]	<0.01
M883I (BS)	3255/10125	0.87[0.77,0.99]	0.03	0.77[0.54,1.10]	0.16	0.90[0.71,1.16]	0.42	0.87[0.71,1.05]	0.15
M883I (BH)	3571/13041	0.88[0.79,0.98]	0.02	0.80[0.58,1.10]	0.17	0.92[0.73,1.15]	0.44	0.84[0.71,0.98]	0.03
M883I (BT)	3870/13084	0.84[0.76,0.92]	<0.01	0.75[0.58,0.95]	0.02	0.89[0.72,1.10]	0.29	0.85[0.71,1.01]	0.07

A: Asians, C: Caucasians, PB: population-based, HB: hospital-based.

CS: cohort study, CCS: case-control study.

BS: based on score (Studies with score ≤6 were excluded).

BH: based on HWE (Studies without HWE were excluded).

BT: based on article type (Master theses were excluded).

CAD: coronary artery disease, IS: ischemic stroke. SS: sample size.

### Quantitative Synthesis

**Figure 2:**
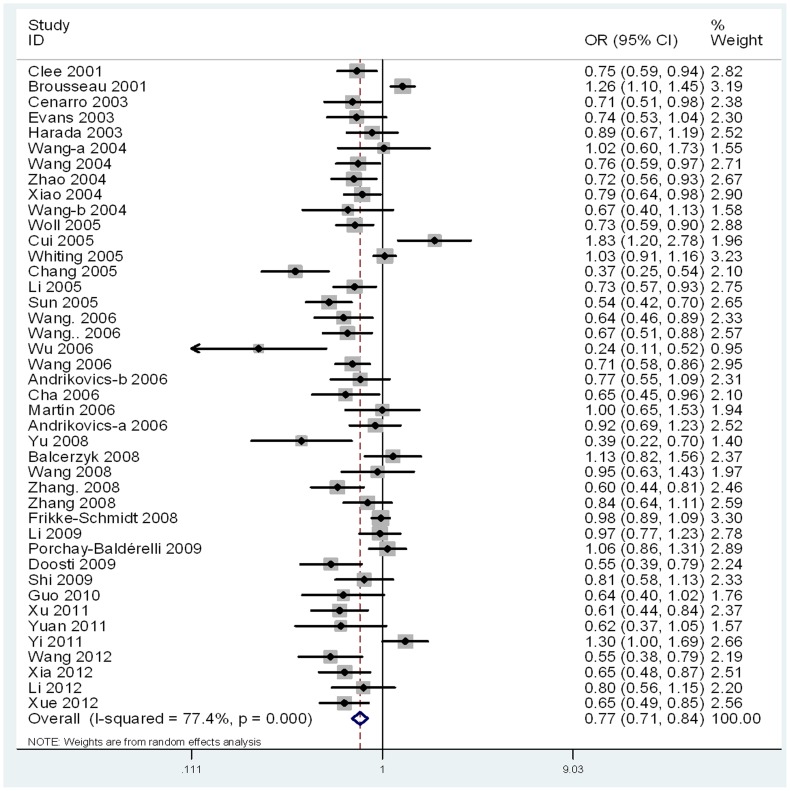
Forest plot for ABCA1 R219K polymorphism and AS risk in the allelic model (K allele vs. R allele).

For the ABCA1 R219K polymorphism, 42 studies were combined. The overall results showed evidence of significant association between the ABCA1 R219K polymorphism and the susceptibility to AS, suggesting that the ABCA1 R219K polymorphism was a protective role for AS (for K allele vs. R allele: OR = 0.77, 95% CI = 0.71–0.84, *P*<0.01; for K/K vs. R/R: OR = 0.60, 95% CI = 0.51–0.71, *P*<0.01; for K/K vs. R/K+R/R: OR = 0.69, 95% CI = 0.60–0.80, *P*<0.01; for K/K+R/K vs. R/R: OR = 0.74, 95% CI = 0.66–0.83, *P*<0.01). In addition, significant associations were also found between this variant and the susceptibility to AS in the Asians group (for K allele vs. R allele: OR = 0.71, 95% CI = 0.64–0.79, *P*<0.01; for K/K vs. R/R: OR = 0.52, 95% CI = 0.42–0.63, *P*<0.01; for K/K vs. R/K+R/R: OR = 0.62, 95% CI = 0.52–0.75, *P*<0.01; for K/K+R/K vs. R/R: OR = 0.66, 95% CI = 0.59–0.74, *P*<0.01), CAD group (for K allele vs. R allele: OR = 0.74, 95% CI = 0.67–0.82, *P*<0.01; for K/K vs. R/R: OR = 0.56, 95% CI = 0.47–0.67, *P*<0.01; for K/K vs. R/K+R/R: OR = 0.64, 95% CI = 0.55–0.74, *P*<0.01; for K/K+R/K vs. R/R: OR = 0.72, 95% CI = 0.64–0.82, *P*<0.01), population-based group (for K allele vs. R allele: OR = 0.76, 95% CI = 0.67–0.85, *P*<0.01; for K/K vs. R/R: OR = 0.58, 95% CI = 0.47–0.72, *P*<0.01; for K/K vs. R/K+R/R: OR = 0.68, 95% CI = 0.56–0.82, *P*<0.01; for K/K+R/K vs. R/R: OR = 0.72, 95% CI = 0.62–0.83, *P*<0.01), hospital-based group (for K allele vs. R allele: OR = 0.81, 95% CI = 0.71–0.92, *P*<0.01; for K/K vs. R/R: OR = 0.65, 95% CI = 0.51–0.82, *P*<0.01; for K/K vs. R/K+R/R: OR = 0.72, 95% CI = 0.59–0.87, *P*<0.01; for K/K+R/K vs. R/R: OR = 0.80, 95% CI = 0.68–0.94, *P*<0.01) and the subgroup of case-control study (for K allele vs. R allele: OR = 0.74, 95% CI = 0.67–0.82, *P*<0.01; for K/K vs. R/R: OR = 0.57, 95% CI = 0.47–0.68, *P*<0.01; for K/K vs. R/K+R/R: OR = 0.67, 95% CI = 0.57–0.78, *P*<0.01; for K/K+R/K vs. R/R: OR = 0.70, 95% CI = 0.62–0.79, *P*<0.01), respectively. The main results of meta-analysis were shown in Table 2, Fig. 2, Fig. S1, Fig. S2 and Fig. S3.

**Figure 3:**
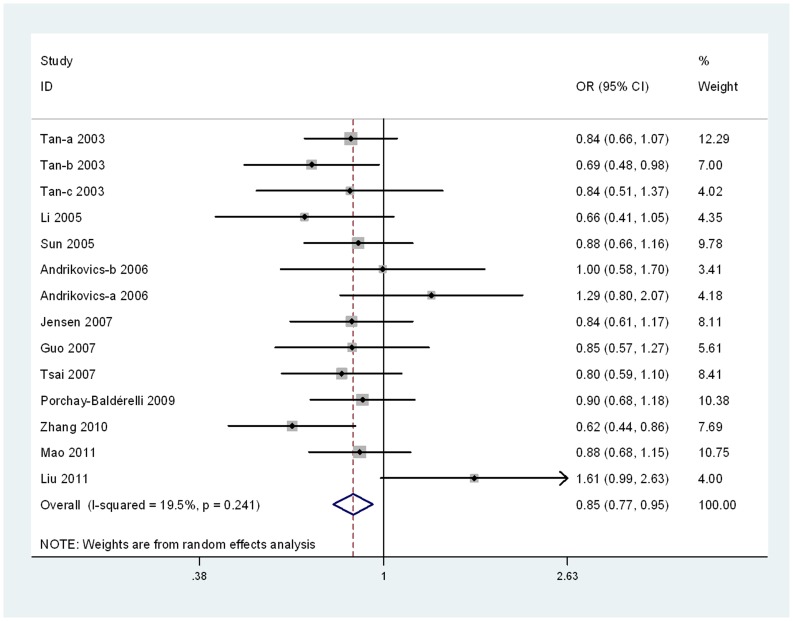
Forest plot for ABCA1 M883I polymorphism and AS risk in the allelic model (I allele vs. M allele).

For the ABCA1 M883I polymorphism, 16 studies were combined. When all 16 studies were pooled into the meta-analysis, there was significant association between the ABCA1 M883I polymorphism and the susceptibility to AS in the allelic model (for I allele vs. M allele: OR = 0.85, 95% CI = 0.77–0.95, *P*<0.01). However, no obvious evidence of association was found in the additive model (for I/I vs. M/M: OR = 0.79, 95% CI = 0.61–1.01, *P* = 0.06), recessive model (for I/I vs. M/I+M/M: OR = 0.92, 95% CI = 0.74–1.13, *P* = 0.42) and dominant model (for I/I+M/I vs. M/M: OR = 0.86, 95% CI = 0.72–1.02, *P* = 0.08). In the subgroup analyses, significant associations were found between this variant and the susceptibility to AS in the Asians group (for I allele vs. M allele: OR = 0.82, 95% CI = 0.72–0.93, *P*<0.01; for I/I vs. M/M: OR = 0.72, 95% CI = 0.54–0.95, *P* = 0.02; for I/I+M/I vs. M/M: OR = 0.75, 95% CI = 0.61–0.91, *P*<0.01), CAD group (for I allele vs. M allele: OR = 0.82, 95% CI = 0.74–0.90, *P*<0.01; for I/I vs. M/M: OR = 0.73, 95% CI = 0.57–0.94, *P* = 0.01; for I/I+M/I vs. M/M: OR = 0.81, 95% CI = 0.68–0.97, *P = *0.02), IS group (for I allele vs. M allele: OR = 1.43, 95% CI = 1.02–2.02, *P = *0.04), population-based group (for I allele vs. M allele: OR = 0.87, 95% CI = 0.78–0.97, *P*<0.01) and the subgroup of case-control study (for I allele vs. M allele: OR = 0.85, 95% CI = 0.75–0.97, *P = *0.01; for I/I+M/I vs. M/M: OR = 0.79, 95% CI = 0.65–0.95, *P = *0.01), respectively. The main results of meta-analysis were shown in Table 2, Fig. 3, Fig. S4, Fig. S5, and Fig. S6.

### Sensitivity Analysis

Sensitivity analyses were conducted to determine whether modification of the inclusion criteria of the meta-analysis affected the final results. The included studies were limited to those conforming to HWE or the high quality studies (NOS score ≥7). In addition, we also performed sensitivity analysis by removing the master thesis. Overall, the corresponding pooled ORs were not materially altered, either for the ABCA1 R219K polymorphism or for M883I polymorphism. This suggested that the overall results of this meta-analysis were statistically robust. The main results of sensitivity analyses were shown in Table 2.

### Heterogeneity Analysis

For the ABCA1 R219K polymorphism, significant heterogeneity existed in the overall comparisons (for allelic model: *P_Q_*<0.01, I^2^ = 77.4%; for additive model: *P_Q_*<0.01, I^2^ = 67.9%; for recessive model: *P_Q_*<0.01, I^2^ = 64.2%; for dominant model: *P_Q_*<0.01, I^2^ = 69.7%). For the ABCA1 M883I polymorphism, significant heterogeneity existed in the dominant model (for *P_Q_*<0.01, I^2^ = 64.3%). In contrast, the allelic model, additive model and recessive model did not present significant heterogeneity (for allelic model: *P_Q_* = 0.24, I^2^ = 19.5%; additive model: *P_Q_* = 0.37, I^2^ = 7.9%; for recessive model: *P_Q_* = 0.75, I^2^ = 0%). To clarify the sources of heterogeneity, we conducted the meta-regression analysis.

For the ABCA1 R219K polymorphism, heterogeneity can be explained by the subtype of atherosclerotic diseases (allelic model: *P_T2_* = 0.013, additive model: *P_T2_* = 0.034, and recessive model: *P_T2_* = 0.017) and study type (dominant model: *P_T4_* = 0.037). According to the results of the meta-regression, we performed subgroup analyses based on the atherosclerotic diseases (CAD and IS) and study type (case-control and cohort study). Heterogeneity was distinctly reduced, although it was still significant in the allelic model (IS group: *P_Q_* = 0.0005, I^2^ = 71%; CAD group: *P_Q_*<0.01, I^2^ = 79%), additive model (IS group: *P_Q_* = 0.001, I^2^ = 69%; CAD group: *P_Q_*<0.01, I^2^ = 68%), recessive model (IS group: *P_Q_* = 0.0008, I^2^ = 70%; CAD group: *P_Q_*<0.01, I^2^ = 59%) and dominant model (CS group: *P_Q_* = 0.14, I^2^ = 42%; CCS group: *P_Q_*<0.01, I^2^ = 66%). For the ABCA1 M883I polymorphism, we did not indentify the sources of heterogeneity by the meta-regression. The main results of meta-regression were shown in Table S3 and Table S4.

### Publication Bias

**Figure 4:**
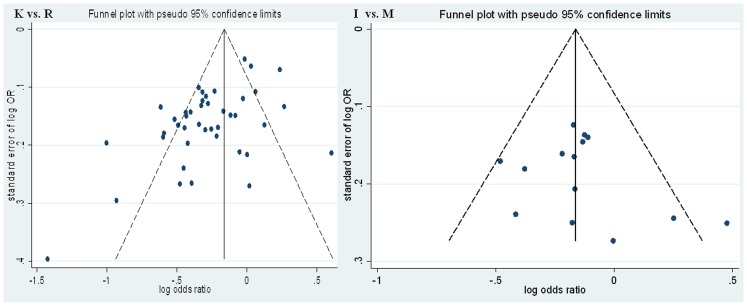
Funnel plots for ABCA1 R219K and M883I polymorphisms and AS risk. K allele vs. R allele for ABCA1 R219K polymorphism. I allele vs. M allele for ABCA1 M883I polymorphism.

Begg’s funnel plot and Egger’s regression test were performed to assess potential publication bias. For the ABCA1 R219K polymorphism, visual inspection of the funnel plot (Fig. 4: K allele vs. R allele) displays asymmetrical distribution of OR estimations, suggesting significant publication bias. In addition, the results of Egger’s regression test also provided evidence for publication bias (*P*<0.01 for allelic model, *P*<0.01 for additive model, *P*<0.01 for recessive model, and *P*<0.01 for dominant model). For the ABCA1 M883I polymorphism, no obvious asymmetry was observed in any genetic model according to the visual assessment of funnel plot (Fig. 4: I allele vs. M allele). Moreover, the results of Egger’s regression test did not provide any statistical evidence for publication bias (*P* = 0.34 for allelic model, *P* = 0.24 for additive model, *P* = 0.10 for recessive model, and *P* = 0.09 for dominant model). In addition to the above analyses, we also calculated the Nfs (for the ABCA1 R219K polymorphism, Nfs = 133 for allelic model, Nfs = 146 for additive model, Nfs = 75 for recessive model, and Nfs = 49 for dominant model; for the ABCA1 M883I polymorphism, Nfs = 50 for allelic model), which reflected the minimum number of non-significant studies that would lead to the P-value to non-significant (*P*>0.05).

## Discussion

To the best of our knowledge, this is the first comprehensive meta-analysis to date investigating the associations between the ABCA1 R219K and M883I polymorphisms and AS risk. Forty-two studies investigating the association between the ABCA1 R219K polymorphism and AS risk were combined [2]–[41], and 16 studies investigating the association between the ABCA1 M883I polymorphism and AS risk were combined [14], [15], [22], [23], [29], [30], [42]–[48]. According to the GRADE approach, the quality of the evidence was very low in the ABCA1 R219K polymorphism. For the ABCA1 M883I polymorphism, GRADE suggested that the quality of the evidence was moderate, except for the dominant model (Low). The overall findings showed that the ABCA1 R219K and M883I polymorphisms may exert an reduced risk effect on AS. For the ABCA1 R219K polymorphism, the risk of developing AS in R allele carriers was 1.30-fold higher than those without R allele. Furthermore, the individuals with K/K genotype had a significantly lower risk for developing AS (for OR = 0.60 in additive model and OR = 0.69 in recessive model) compared to those with R/K genotype and R/R genotype. As for the ABCA1 M883I polymorphism, the combined evidence showed that the individuals with I allele had a significantly lower risk for developing AS (for OR = 0.85 in allelic model) compared to those without I allele. Therefore, it is reasonable to assume that the ABCA1 R219K K allele and M883I I allele are the protective factors for the development of AS.

To make a more comprehensive analysis, subgroup analyses were performed based on ethnicity, atherosclerotic disease, source of controls and study type. For the ABCA1 R219K polymorphism, significant associations were found between this variant and the susceptibility to AS in the Asians group, CAD group, population-based group, hospital-based group and the subgroup of case-control study, respectively. These results further strengthened the conclusion that the ABCA1 R219K K allele was a protective factor for AS. However, we did not find significant association between this variant and AS risk in Caucasians group, IS group and the subgroup of cohort study. For the ABCA1 M883I polymorphism, significant associations were found between this variant and the susceptibility to AS in the Asians group, CAD group, IS group, population-based group and the subgroup of case-control study, respectively. Similarly, no significant association was found between the ABCA1 M883I polymorphism and AS risk in Caucasians group and the subgroup of cohort study. Moreover, we also did not find significant association between this variant and AS risk in hospital-based group. One potential explanation is that controls in the hospital-based group may not truly represent the underlying source populations because the ABCA1 M883I polymorphism has been reported to be associated with various diseases. For instance, in 2004, Katzov et al. found that the ABCA1 polymorphism were involved in the process of cholesterol metabolism of the brain and may affect the risk of Alzheimer’s disease [62]. In 2007, Kitjaroentham et al. indicated that the presence of ABCA1 polymorphism could be a risk factor for overweight/obesity among Thai males [63]. However, there was a noteworthy result in the IS group which suggested the ABCA1 M883I polymorphism was associated with an increased AS risk. This finding was inconsistent with the overall results, which may affect the interpretation of the final results. Throughout the IS group, it should be noted that the sample size in this group was really small (334 IS cases and 205 controls), which could increase the probability of false positives or false negatives. Therefore, this result should be interpreted with caution.

In the subgroup analysis based on ethnicity, we only obtained the positive result in the Asians. For Caucasians, we did not find significant association between the ABCA1 R219K and M883I polymorphisms and AS risk. In fact, the minor allele frequencies (MAF) of ABCA1 R219K and M883I are low among utah residents with Northern and Western European ancestry (CEU) (MAF = 0.210 and 0.133 in HapMap CEU) [64]. However, the ABCA1 R219K and M883I polymorphisms are common among Han Chinese in Beijing, China (CHB) and Japanese in Tokyo, Japan (JPT) (MAF = 0.453/0.434 and 0.255/0.438, respectively) [64]. Compared with the overall results, the inconsistent results of Caucasians group may be partly due to genetic diversity among ethnicities. Moreover, as AS is a multifactorial disease, in addition to genetic factors, environmental factors also play an important role in AS etiology. Thus, this discrepancy may also be caused by varied geographic distribution, linked to climate, diet, lifestyle and economic status.

Considering the studies without HWE or with low NOS score may influence the overall results, subsequent sensitivity analyses restricted to the studies with HWE or high NOS score were performed. Although the corresponding pooled OR was materially altered in the dominant model (OR = 0.84, 95% CI = 0.71–0.98, *P* = 0.03) of the ABCA1 M883I polymorphism (Based on HWE), this result further strengthened the conclusion that the ABCA1 M883I polymorphism was a protective factor for AS. As for the ABCA1 R219K polymorphism, the corresponding pooled ORs were not materially altered in all comparisons. These results suggested that the studies without HWE should be considered as a factor influencing the overall results.

Significant heterogeneity existed in the present meta-analysis, either for the ABCA1 R219K polymorphism or for the ABCA1 M883I polymorphism. Heterogeneity should not be ignored and should be carefully factored in the interpretation of the finally results [65]. For the ABCA1 R219K polymorphism, the heterogeneity can be explained by the subtype of atherosclerotic diseases (CAD and IS) and study type (case-control and cohort study). For the ABCA1 M883I polymorphism, we did not indentify the sources of heterogeneity by the meta-regression. Common reasons of heterogeneity may include diversity in design, measurement errors, difference of ethnicity, or the interaction with other risk factors. Due to lack of original data, we did not perform further meta-regression analysis by the other factors.

For better interpreting the results, some limitations of this meta-analysis should be acknowledged. Firstly, there was significant heterogeneity in this meta-analysis. Heterogeneity may affect the precision of overall results, despite the use of appropriate meta-analytic techniques with random-effects model. Secondly, in the subgroup analyses, the sample sizes in some subgroup, such as the IS group and the hospital-based group of the ABCA1 M883I polymorphism, were relatively small, not having enough statistical power to explore the real association. Thirdly, publication bias was a potential problem that may bias the present results. Due to language restriction, some inevitable publication bias may exist in the present meta-analysis. Moreover, AS is a complex disease, involving potential interactions among gene-gene and gene-environment. A single gene polymorphism is insufficient to provide the complete explanation of genetic risk for AS. However, many eligible studies included in this meta-analysis did not consider the environmental factors.

In conclusion, the present meta-analysis suggested that the ABCA1 R219K and M883I polymorphisms were associated with the susceptibility to AS, especially in Asians. However, the result should be interpreted with caution because of its limitations. Further studies with large sample size, especially with the consideration of gene-gene and gene-environment interactions, will be needed to confirm our findings.

## Supporting Information

Figure S1 Forest plot for ABCA1 R219K polymorphism and AS risk in the additive model (K/K vs. R/R).
(TIF)

Figure S2 Forest plot for ABCA1 R219K polymorphism and AS risk in the rssive model (K/K vs. R/K+R/R).
(TIF)

Figure S3 Forest plot for ABCA1 R219K polymorphism and AS risk in the dominant model (K/K+R/K vs. R/R).
(TIF)

Figure S4 Forest plot for ABCA1 M883I polymorphism and AS risk in the additive model (I/I vs. M/M).
(TIF)

Figure S5 Forest plot for ABCA1 M883I polymorphism and AS risk in the recessive model (I/I vs. M/I+M/M).
(TIF)

Figure S6 Forest plot for ABCA1 M883I polymorphism and AS risk in the dominant model (I/I+M/I vs. M/M).
(TIF)

Table S1 MOOSE checklist.
(DOC)

Table S2 GRADE profile evidence of the included studies.
(DOC)

Table S3 The meta-regression results for the association of the ABCA1 R219K polymorphism and AS.
(DOC)

Table S4 The meta-regression results for the association of the ABCA1 M883I polymorphism and AS.
(DOC)

Checklist S1 PRISMA 2009 checklist.
(DOC)
