# Supplementary figures and images for: Influence of ATP-Binding Cassette Transporter 1 R219K and M883I Polymorphisms on Development of Atherosclerosis: A Meta-Analysis of 58 Studies
Source: PLoS One. 2014 Jan 23;9(1):e86480. doi: 10.1371/journal.pone.0086480 (PMC3900558; doi:10.1371/journal.pone.0086480)

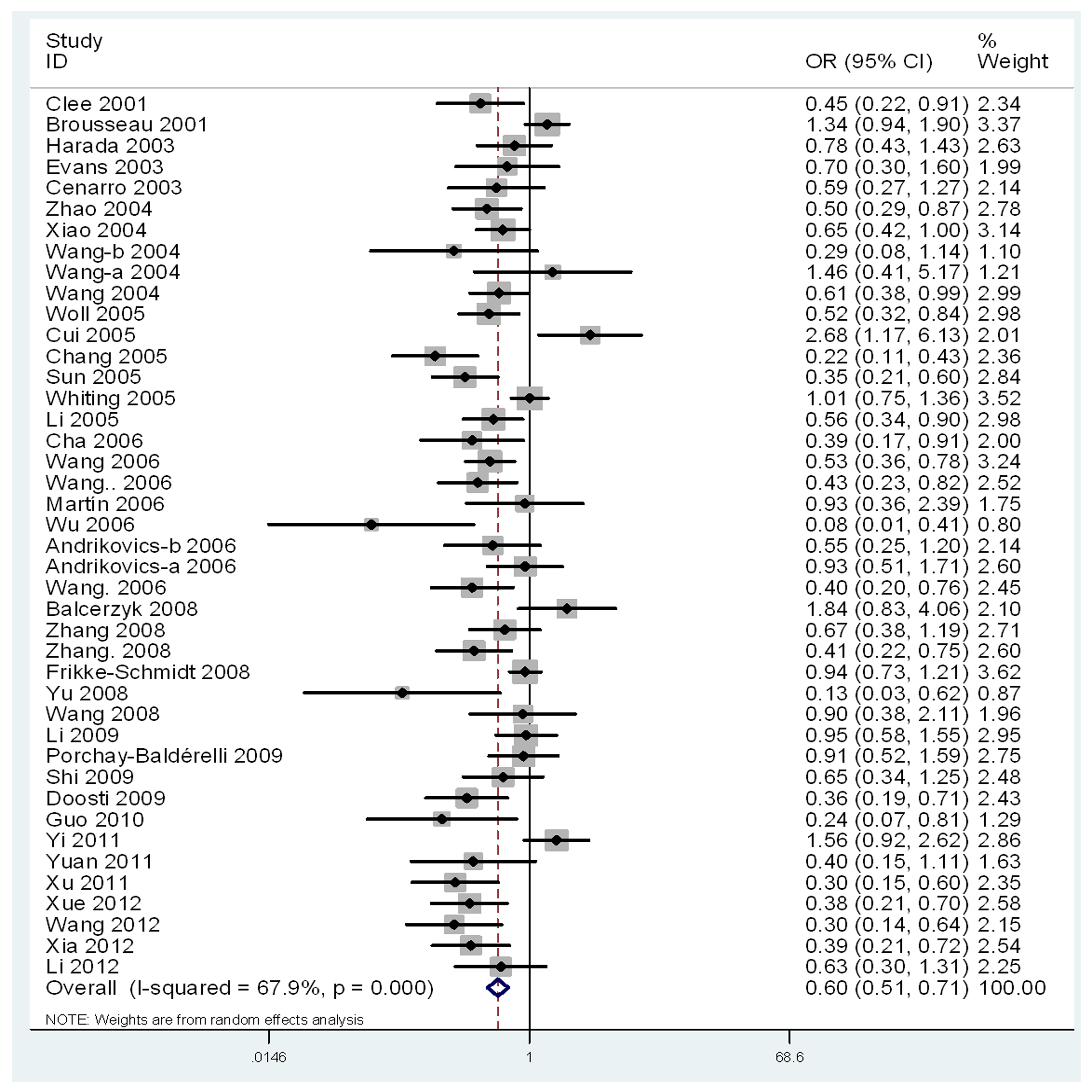

Supplement: Figure S1 — Forest plot for ABCA1 R219K polymorphism and AS risk in the additive model (K/K vs. R/R). (TIF) [file pone.0086480.s001.tif]

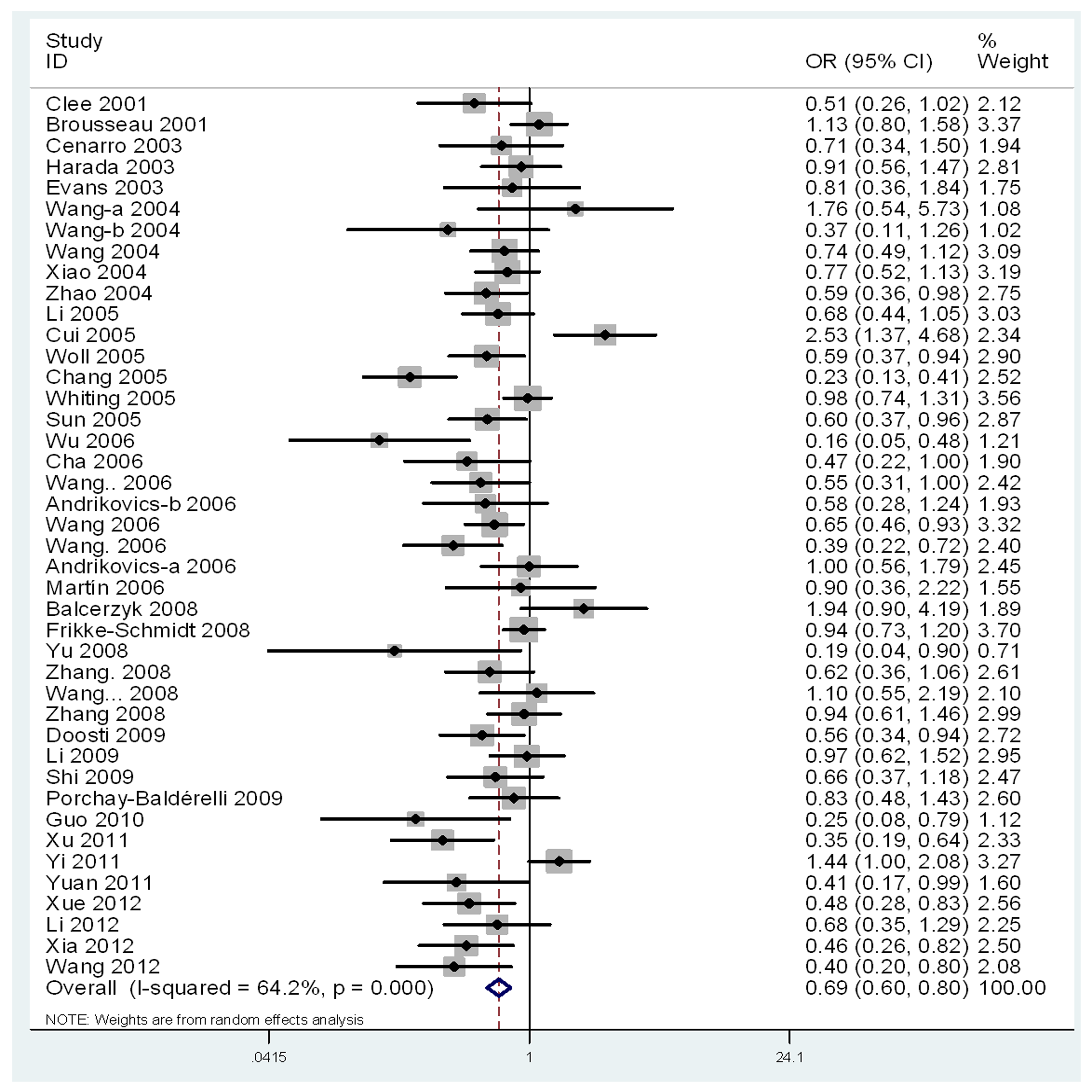

Supplement: Figure S2 — Forest plot for ABCA1 R219K polymorphism and AS risk in the rssive model (K/K vs. R/K+R/R). (TIF) [file pone.0086480.s002.tif]

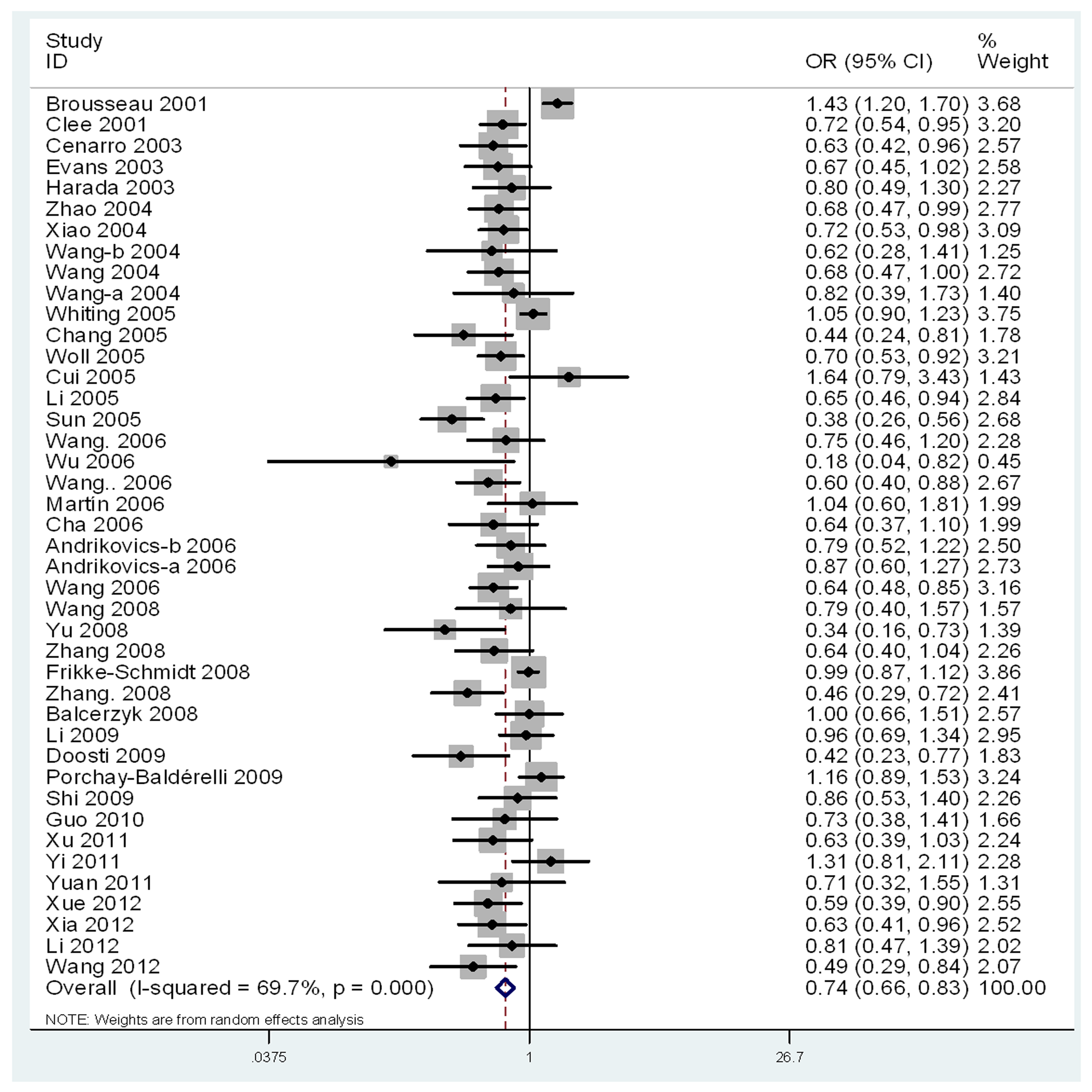

Supplement: Figure S3 — Forest plot for ABCA1 R219K polymorphism and AS risk in the dominant model (K/K+R/K vs. R/R). (TIF) [file pone.0086480.s003.tif]

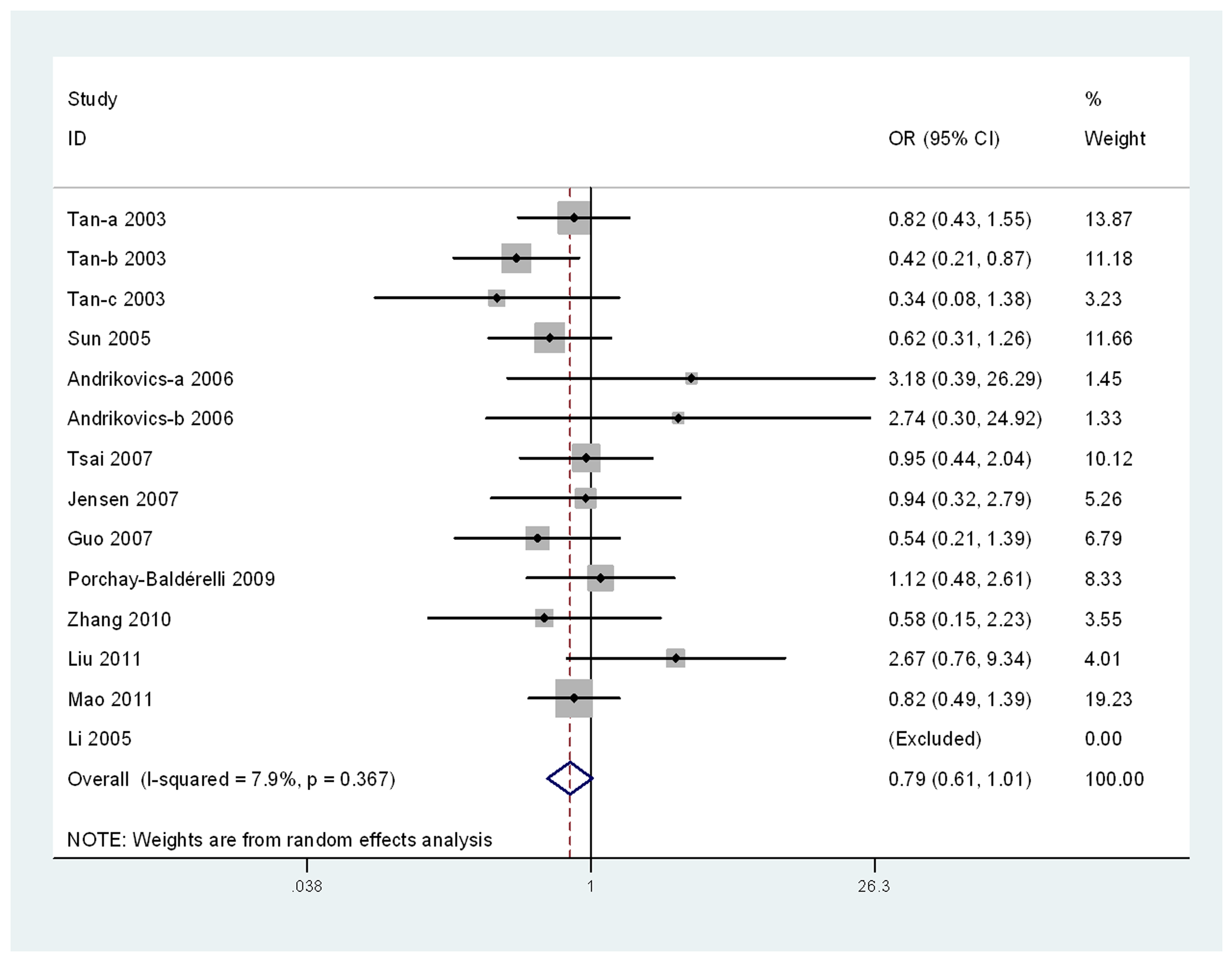

Supplement: Figure S4 — Forest plot for ABCA1 M883I polymorphism and AS risk in the additive model (I/I vs. M/M). (TIF) [file pone.0086480.s004.tif]

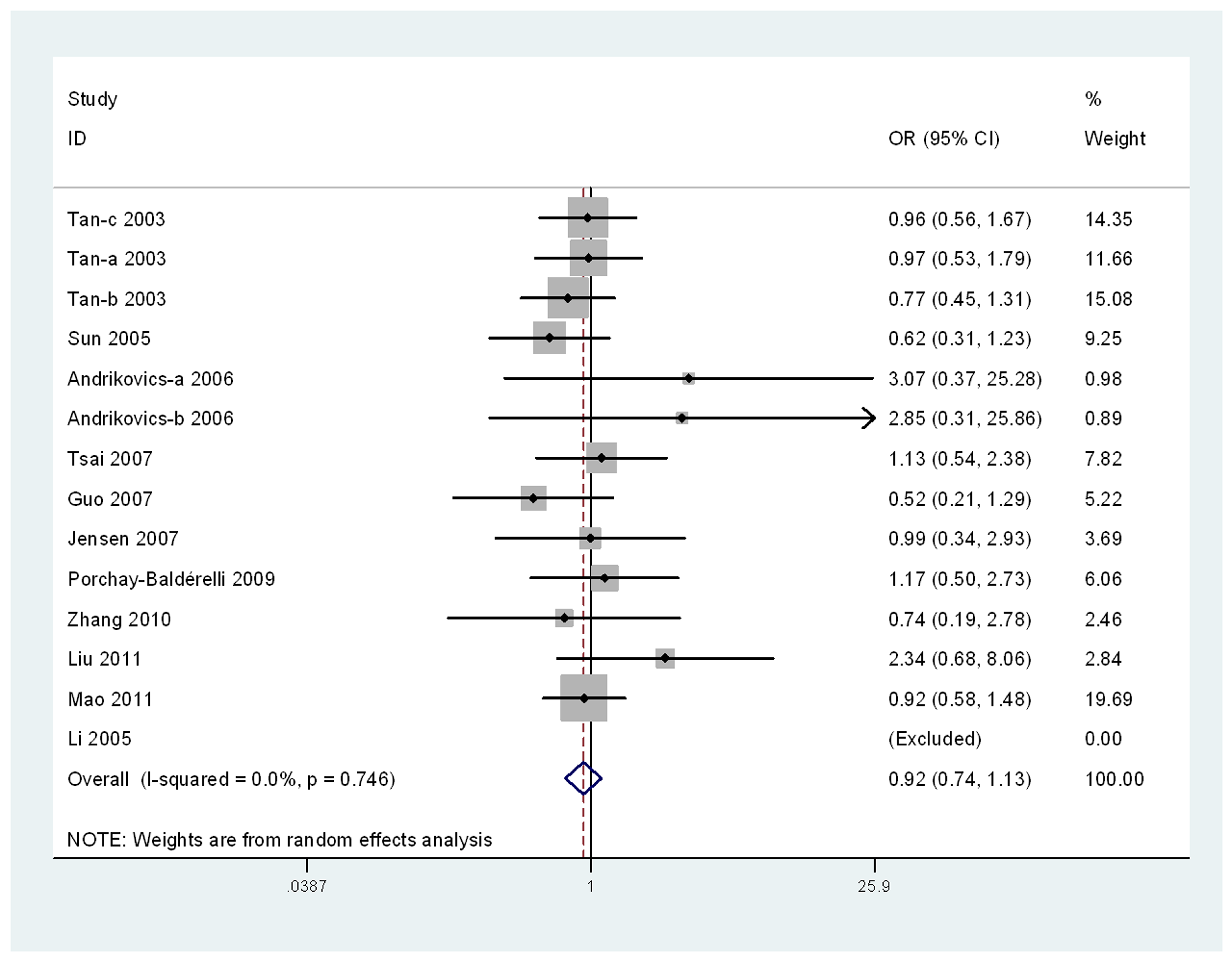

Supplement: Figure S5 — Forest plot for ABCA1 M883I polymorphism and AS risk in the recessive model (I/I vs. M/I+M/M). (TIF) [file pone.0086480.s005.tif]

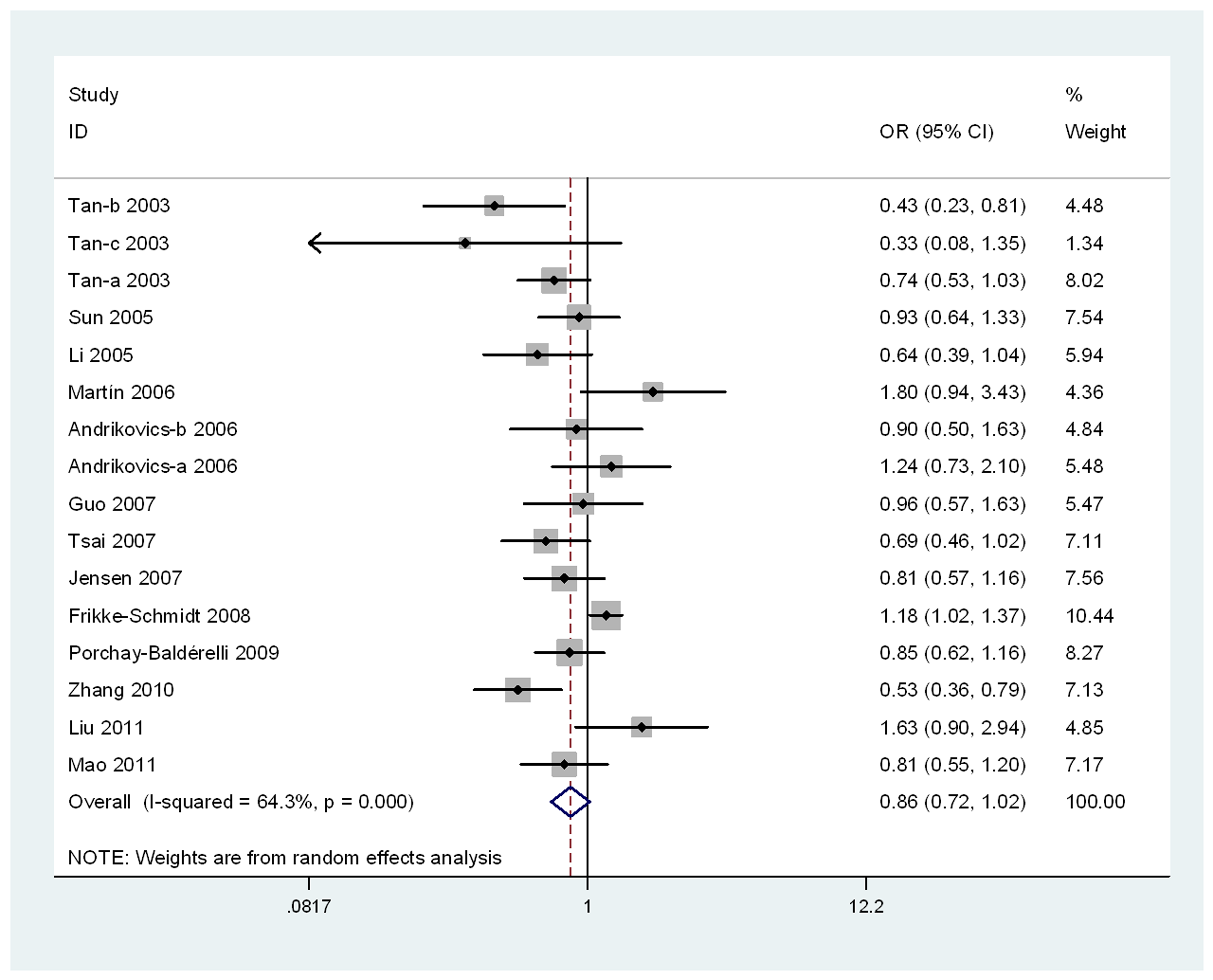

Supplement: Figure S6 — Forest plot for ABCA1 M883I polymorphism and AS risk in the dominant model (I/I+M/I vs. M/M). (TIF) [file pone.0086480.s006.tif]
