# Supplementary material for: Influence of ATP-Binding Cassette Transporter 1 R219K and M883I Polymorphisms on Development of Atherosclerosis: A Meta-Analysis of 58 Studies
Source: PLoS One. 2014 Jan 23;9(1):e86480. doi: 10.1371/journal.pone.0086480 (PMC3900558; doi:10.1371/journal.pone.0086480)
Supplement: Table S1 — MOOSE checklist. (DOC) [file pone.0086480.s007.doc]

**Table S1 MOOSE Checklist**

| **Criteria** | | **Brief description of how the criteria were handled in the meta-analysis** |
| --- | --- | --- |
| **Reporting of background should include** | |  |
| √ | Problem definition | ABCA1 R219K and M883I polymorphisms are closely associated with various diseases including CAD, IS and MI. However, the associations between ABCA1 R219K and M883I polymorphisms and susceptibility to AS were inconsistent in previous studies. |
| √ | Hypothesis statement | It is likely that ABCA1 R219K and M883I polymorphisms may influence the susceptibility of AS. |
| √ | Description of study outcomes | Atherosclerosis |
| √ | Type of exposure or intervention used | RK/MI, RR/MM, RK+RR/MI+MM genotypes or K/I allele |
| √ | Type of study designs used | Published case-control, nested case-control or cohort designs studies. |
| √ | Study population | No restriction. |
| **Reporting of search strategy should include** | |  |
| √ | Qualifications of searchers | Investigators include experts in atherosclerotic diseases and qualified graduate students. All of the investigators have received training in literature research, statistics and evidence-based medicine. |
| √ | Search strategy, including time period included in the synthesis and keywords | We selected possibly relevant articles in PubMed, Embase, Web of Science, Medline, Cochrane database, Clinicaltrials.gov, Current Controlled Trials, Chinese Clinical Trial Registry, CBMdisc, CNKI, Google Scholar and Baidu Library (last search was update on November 15, 2013) with search strategy: (“ATP-binding cassette transporter A1” OR “ATP-binding cassette sub-family A member-1” OR “ABCA1”) AND (“polymorphism” OR “mutation” OR “variant” OR “variation” OR “genotype”) AND (“coronary artery disease” OR “CAD” OR “coronary heart disease” OR “CHD” OR “myocardial infarction” OR “MI” OR “ischemic cardiovascular disease” OR “ischemic cardiovascular events” OR “ischemic stroke” OR “IS” OR “cerebrovascular disease” OR “ischemic cerebrovascular events” OR “cerebral infarction” OR “cerebral ischemia” OR “brain infarction” OR “carotid artery stenosis” OR “CAAD” OR “transient ischemic attack” OR “TIA” OR “peripheral Arterial Disease” OR “PAD” OR “peripheral artery occlusive disease” OR “PAOD” OR “renal artery stenosis” OR “RAS” OR “retinal artery occlusion” OR “RAO” OR “aortic aneurysm” OR “atherosclerosis”). |
| √ | Databases and registries searched | PubMed, Embase, Web of Science, Medline, Cochrane database, Clinicaltrials.gov, Current Controlled Trials, Chinese Clinical Trial Registry, CBMdisc, CNKI, Google Scholar and Baidu Library |
| √ | Search software used, name and version, including special features | We did not employ any search software. |
|  | Use of hand searching | Other relevant studies were identiﬁed by hand-searching the references of included articles identiﬁed by electronic search and the abstracts presented at related scientific societies meetings. |
| √ | List of citations located and those excluded, including justifications | Literature search and selection process are outlined in the flow diagram. The reasons for exclusion were listed in the flow diagram and explained in result section. |
|  | Method of addressing articles published in languages other than English | The search was limited to English and Chinese language papers. |
| √ | Method of handling abstracts and unpublished studies | We first examined if overlap existed and excluded overlapped studies. We only included published case-control, nested case-control or cohort designs studies. |
| √ | Description of any contact with authors | If necessary data were not reported in the primary manuscripts, we contacted the corresponding authors by email to request the missing data. |
| **Reporting of methods should include** | |  |
| √ | Description of relevance or appropriateness of studies assembled for assessing the hypothesis to be tested | Detailed inclusion and exclusion criteria were described in the methods. |
| √ | Rationale for the selection and coding of data | We only used the crude ORs and 95%CIs for meta-analysis. If the studies did not provide crude ORs and 95%CIs, we calculated the ORs and 95%CIs by the total numbers of cases and controls, and frequencies of R219K and M883I polymorphisms in cases and controls. |
| √ | Assessment of confounding | NOS rating system was used to assess the confounder. Subgroup analyses were performed and sensitivity analyses were also performed. |
| √ | Assessment of study quality, including blinding of quality assessors; stratification or regression on possible predictors of study results | We assessed the methodological qualities of included studies by the description of study population, the set of controls and cases and related statistical methods. We carried out sensitivity analysis. |
| √ | Assessment of heterogeneity | Heterogeneity was assessed by the Q-test and I^2^ statistic, P<0.10 and I^2^>50% indicated evidence of heterogeneity. |
| √ | Description of statistical methods in sufficient detail to be replicated | Methods of heterogeneity test, quantitative synthesis, assessments of publication bias, sensitivity analyses were reported in detail in the methods section. |
| √ | Provision of appropriate tables and graphics | We provided flow chart to explain literature searching and selection (Figure 1); forest plots for the total analysis, (Figure 2, Figure 3, Figure S1, Figure S2, Figure S3, Figure S4, Figure S5, Figure S6); study characteristics and allele/genotype frequencies (Table 1). |
| **Reporting of results should include** | |  |
|  | Graph summarizing individual study estimates and overall estimate | Graph summarizing individual study estimates and overall estimate are presenting in Figure 2, Figure 3, Figure S1, Figure S2, Figure S3, Figure S4, Figure S5 and Figure S6. |
| √ | Table giving descriptive information for each study included | Descriptive information for each study included was provided in Table 1. |
| √ | Results of sensitivity testing | The results of sensitivity analysis were described in results section. Table 2 provided detailed results for the sensitivity analyses. |
| √ | Indication of statistical uncertainty of findings | The results of heterogeneity test, pooled ORs, 95% confidence intervals and *P* value for *Z* test were presented with all pooled analyses. |
| **Reporting of discussion should include** | |  |
| √ | Quantitative assessment of bias | We evaluated the publication bias by funnel plots, egger’s test and Nfs. |
| √ | Justification for exclusion | Based on our preliminary search criteria, a total of 234 publications were eligible. Among these studies, 12 studies were review articles. 170 studies did not report the associations between the ABCA1 R219K and M883I polymorphisms and AS risk, or reported other polymorphisms rather than the ABCA1 R219K and M883I polymorphisms. Furthermore, five studies were meta-analysis. |
| √ | Assessment of quality of included studies | We discussed the results of sensitivity analyses and described the limitations of included studies. |
| **Reporting of conclusions should include** | |  |
| √ | Consideration of alternative explanations for observed results | We discussed that potential unmeasured confounders and explained the limitations of this meta-analysis. We reminded readers that caution should be made when interpreting this meta-analysis. |
| √ | Generalization of the conclusions | Our meta-analysis suggested that the ABCA1 R219K and M883I polymorphisms were associated with the susceptibility to AS. |
| √ | Guidelines for future research | Larger sample-size studies with homogeneous AS patients and well-matched controls are required. |
| √ | Disclosure of funding source | This study was supported by grants from Natural Science Foundation Project of CQ CSTC (CSTC2012JJJQ10003 to Li-li Zhang) and National Natural Science Foundation of China ( NSFC 81271282 to Jing-cheng Li). |
