# Supplementary material for: Influence of ATP-Binding Cassette Transporter 1 R219K and M883I Polymorphisms on Development of Atherosclerosis: A Meta-Analysis of 58 Studies
Source: PLoS One. 2014 Jan 23;9(1):e86480. doi: 10.1371/journal.pone.0086480 (PMC3900558; doi:10.1371/journal.pone.0086480)
Supplement: Table S2 — GRADE profile evidence of the included studies. (DOC) [file pone.0086480.s008.doc]

**Table S2 GRADE profile evidence of the included studies**

| Position | Category | No. of  studies | Quality assessment | | | | |  | Quality |  | Importance |
| --- | --- | --- | --- | --- | --- | --- | --- | --- | --- | --- | --- |
|  |  |  | Risk of bias | Inconsistency | Indirectness | Imprecision | Publication bias |  |  |  |  |
| R219K | Allelic model | 42 | Serious^a^ | Serious^b^ | No | No | Strongly suspected^c^ |  | ⊕○○○ (Very low) |  | Critical |
|  | Additive model | 42 | Serious^a^ | Serious^b^ | No | No | Strongly suspected^c^ |  | ⊕○○○ (Very low) |  | Critical |
|  | Recessive model | 42 | Serious^a^ | Serious^b^ | No | No | Strongly suspected^c^ |  | ⊕○○○ (Very low) |  | Critical |
|  | Dominant model | 42 | Serious^a^ | Serious^b^ | No | No | Strongly suspected^c^ |  | ⊕○○○ (Very low) |  | Critical |
| M883I | Allelic model | 16 | Serious^a^ | No | No | No | Undetected |  | ⊕⊕⊕○ (Moderate) |  | Critical |
|  | Additive model | 16 | Serious^a^ | No | No | No | Undetected |  | ⊕⊕⊕○ (Moderate) |  | Critical |
|  | Recessive model | 16 | Serious^a^ | No | No | No | Undetected |  | ⊕⊕⊕○ (Moderate) |  | Critical |
|  | Dominant model | 16 | Serious^a^ | Serious^b^ | No | No | Undetected |  | ⊕⊕○○ (Low) |  | Critical |

**^a^ Evidence limited by study design and implementation (observational study).**

**^b^ High heterogeneity existed in the comparison.**

**^c^ Significant publication bias existed in the study.**
