# Supplementary material for: Influence of ATP-Binding Cassette Transporter 1 R219K and M883I Polymorphisms on Development of Atherosclerosis: A Meta-Analysis of 58 Studies
Source: PLoS One. 2014 Jan 23;9(1):e86480. doi: 10.1371/journal.pone.0086480 (PMC3900558; doi:10.1371/journal.pone.0086480)
Supplement: Table S3 — The meta-regression results for the association of the ABCA1 R219K polymorphism and AS. (DOC) [file pone.0086480.s009.doc]

**Table S3 The meta-regression results for the association of the ABCA1 R219K polymorphism and AS.**

|  |  | Coefficient | Standard Error | T value | P value | 95% Confidence Interval |
| --- | --- | --- | --- | --- | --- | --- |
| Allelic model | T1 | -0.1249423 | 0.0880553 | -1.42 | 0.164 | -0.3033593～0.0534747 |
|  | T2 | 0.3110728 | 0.1191338 | 2.61 | 0.013 | 0.0696847～0.5524609 |
|  | T3 | 0.0464336 | 0.1089563 | 0.43 | 0.672 | -0.1743329～0.2672001 |
|  | T4 | -0.2477454 | 0.1397799 | -1.77 | 0.085 | -0.5309663～0.0354755 |
|  | _cons | -0.0052128 | 0.3789059 | -0.01 | 0.989 | -0.7729491～0.7625235 |
| Additive model | T1 | -0.2131065 | 0.1705862 | -1.25 | 0.219 | -0.5587469～0.132534 |
|  | T2 | 0.4915824 | 0.2227904 | 2.21 | 0.034 | 0.0401661～0.9429988 |
|  | T3 | 0.0911225 | 0.2114671 | 0.43 | 0.669 | -0.3373506～0.5195956 |
|  | T4 | -0.394954 | 0.2710523 | -1.46 | 0.154 | -0.9441582～0.1542502 |
|  | _cons | -0.1052261 | 0.7338733 | -0.14 | 0.887 | -1.592195～1.381742 |
| Recessive model | T1 | -0.1418231 | 0.1527045 | -0.93 | 0.359 | -0.4512317～0.1675856 |
|  | T2 | 0.4867006 | 0.1938168 | 2.51 | 0.017 | 0.0939906～0.8794106 |
|  | T3 | 0.0941193 | 0.1857986 | 0.51 | 0.615 | -0.2823444～0.4705829 |
|  | T4 | -0.2549427 | 0.2408364 | -1.06 | 0.297 | -0.7429235～0.2330381 |
|  | _cons | -0.350622 | 0.6455358 | -0.54 | 0.590 | -1.658602～0.9573577 |
| Dominant model | T1 | -0.1257158 | 0.0949073 | -1.32 | 0.193 | -0.3180164～0.0665847 |
|  | T2 | 0.1928135 | 0.1352702 | 1.43 | 0.162 | -0.0812699～0.4668968 |
|  | T3 | 0.0190183 | 0.1259446 | 0.15 | 0.881 | -0.2361698～0.2742063 |
|  | T4 | -0.3280624 | 0.1516616 | -2.16 | 0.037 | -0.635358～-0.0207668 |
|  | _cons | 0.2675614 | 0.4249075 | 0.63 | 0.533 | -0.5933829～1.128506 |

**Coefficient: regression coefficient ; cons: constant item.**

**T1: ethnicity (Caucasians and Asians); T2: atherosclerotic diseases (CAD and IS);**

**T3: source of controls (population-based studies and hospital-based studies);**

**T4: study type (case-control and cohort study).**
