# Supplementary material for: Influence of ATP-Binding Cassette Transporter 1 R219K and M883I Polymorphisms on Development of Atherosclerosis: A Meta-Analysis of 58 Studies
Source: PLoS One. 2014 Jan 23;9(1):e86480. doi: 10.1371/journal.pone.0086480 (PMC3900558; doi:10.1371/journal.pone.0086480)
Supplement: Table S4 — The meta-regression results for the association of the ABCA1 M883I polymorphism and AS. (DOC) [file pone.0086480.s010.doc]

**Table S4 The meta-regression results for the association of the ABCA1 M883I polymorphism and AS.**

|  |  | Coefficient | Standard Error | T value | P value | 95% Confidence Interval |
| --- | --- | --- | --- | --- | --- | --- |
| Dominant model | T1 | -0.0369609 | 0.2931339 | -0.13 | 0.902 | -0.6821442～0.6082224 |
|  | T2 | 0.6252248 | 0.2930547 | 2.13 | 0.056 | -0.0197842～1.270234 |
|  | T3 | -0.1672294 | 0.1727374 | -0.97 | 0.354 | -0.5474218～0.2129629 |
|  | T4 | -0.3721186 | 0.3096942 | -1.20 | 0.255 | -1.053751～0.3095138 |
|  | _cons | 0.0501382 | 0.4462436 | 0.11 | 0.913 | -0.9320374～1.032314 |

**Coefficient: regression coefficient ; cons: constant item.**

**T1: ethnicity (Caucasians and Asians); T2: atherosclerotic diseases (CAD and IS);**

**T3: source of controls (population-based studies and hospital-based studies);**

**T4: study type (case-control and cohort study).**
