# Supplementary material for: Influence of ATP-Binding Cassette Transporter 1 R219K and M883I Polymorphisms on Development of Atherosclerosis: A Meta-Analysis of 58 Studies
Source: PLoS One. 2014 Jan 23;9(1):e86480. doi: 10.1371/journal.pone.0086480 (PMC3900558; doi:10.1371/journal.pone.0086480)
Supplement: Checklist S1 — PRISMA 2009 checklist. (DOC) [file pone.0086480.s011.doc]

| **Section/topic** | **#** | **Checklist item** | **Reported on page #** |
| --- | --- | --- | --- |
| **TITLE** | | |  |
| Title | 1 | Influence of ATP-binding cassette transporter 1 R219K and M883I polymorphisms on development of atherosclerosis: A meta-analysis of 58 studies | Title |
| **ABSTRACT** | | |  |
| Structured summary | 2 | **Background:** Numerous epidemiological studies have evaluated the associations between ATP-binding cassette transporter 1 (ABCA1) R219K (rs2230806) and M883I (rs4149313) polymorphisms and atherosclerosis (AS), but results remain controversial. The purpose of the present study is to investigate whether these two polymorphisms confer signiﬁcant susceptibility to AS using a meta-analysis.  **Methods:** PubMed, Embase, Web of Science, Cochrane database, Clinicaltrials.gov, Current Controlled Trials, Chinese Clinical Trial Registry, CBMdisc, CNKI and Google Scholar were searched to get the genetic association studies. All statistical analyses were done with Stata 11.0.  **Results:** Forty-seven articles involving 58 studies were included in the final meta-analysis. For the ABCA1 R219K polymorphism, 42 studies involving 12551 AS cases and 19548 controls were combined showing significant association between this variant and AS risk (for K allele vs. R allele: OR = 0.77, 95% CI = 0.71-0.84, p < 0.01; for K/K vs. R/R: OR = 0.60, 95% CI = 0.51-0.71, p < 0.01; for K/K vs. R/K+R/R: OR = 0.69, 95% CI = 0.60-0.80, p < 0.01; for K/K+R/K vs. R/R: OR = 0.74, 95% CI = 0.66-0.83, p < 0.01). For the ABCA1 M883I polymorphism, 16 studies involving 4224 AS cases and 3462 controls were combined. There was also significant association between the variant and AS risk (for I allele vs. M allele: OR = 0.85, 95% CI = 0.78-0.93, p < 0.01).  **Conclusions:** The present meta-analysis suggests that the ABCA1 R219K and M883I polymorphisms are associated with the susceptibility to AS. However, due to the high heterogeneity in the meta-analysis, the results should be interpreted with caution. | Abstract |
| **INTRODUCTION** | | |  |
| Rationale | 3 | It is widely accepted that a low level of plasma high-density lipoprotein cholesterol (HDL-C) is a major risk factor for atherosclerotic diseases in the general population [49]. ABCA1, a member of the ATP-binding cassette family，has been implicated in the HDL formation and the cholesterol efﬂux [50]. Previous studies have reported that the mutations in ABCA1 are responsible for two forms of heritable HDL disorders, namely Tangier disease (Homozygosity) and familial hypoalphalipoproteinemia (heterozygosity) [51, 52]. Given the crucial role of ABCA1 in the HDL formation and the cholesterol efﬂux, the mutations in ABCA1 may also play a significant role in the development of atherosclerotic diseases. Recently, a number of molecular epidemiological studies have been done to evaluate the associations between the ABCA1 gene polymorphisms (such as R219K, M883I, C69T, V825I, R1587K, V771M and -565C/T) and the risk of atherosclerotic diseases [2-48]. However, results of different studies have been inconsistent, possibly due to small sample sizes in individual studies. | Introduction |
| Objectives | 4 | Recently, a number of molecular epidemiological studies have been done to evaluate the associations between the ABCA1 gene polymorphisms (such as R219K, M883I, C69T, V825I, R1587K, V771M and -565C/T) and the risk of atherosclerotic diseases [2-48]. However, results of different studies have been inconsistent, possibly due to small sample sizes in individual studies. In 2011, Ma et al. performed a meta-analysis to evaluate the association between the ABCA1 R219K polymorphism and coronary artery disease (CAD), and demonstrated that the ABCA1 R219K polymorphism was a protective role for CAD both in Asians and Caucasians [53]. In 2012, Li et al. also conducted a meta-analysis to evaluate the association of this variant with CAD [54]. However, they just found the ABCA1 R219K polymorphism was a protective factor in Asians, but not in Caucasians. Considering the above two meta-analyses only focused on the association of ABCA1 R219K polymorphism with the single atherosclerotic disease, we therefore performed this meta-analysis of all the studies available now to derive a more precise estimation of the associations between ABCA1 R219K and M883I polymorphisms and overall AS risk. | Introduction |
| **METHODS** | | |  |
| Protocol and registration | 5 | No protocol and registration. |  |
| Eligibility criteria | 6 | To be included in the present meta-analysis, the studies had to comply with the following major criteria: (1) case-control or cohort studies evaluating the associations between the ABCA1 R219K and M883I polymorphisms and AS risk; (2) sufficient published data for calculating odds ratios (ORs) with their 95% confidence intervals (CIs); (3) not republished data. | Inclusion and exclusion criteria |
| Information sources | 7 | Eligible literatures published before the end of June 2013 were identified by the search of PubMed, Embase, Web of Science, Cochrane database, Clinicaltrials.gov, Current Controlled Trials, Chinese Clinical Trial Registry, CBMdisc, CNKI and Google Scholar. In addition, all references cited were reviewed to identify additional studies. | Literature search |
| Search | 8 | Following Medical Subject Heading (MeSH) terms and/or text words were used for searching: (“ATP-binding cassette transporter A1” OR “ATP-binding cassette sub-family A member-1” OR “ABCA1”) AND (“polymorphism” OR “mutation” OR “variant” OR “variation” OR “genotype”) AND (“coronary artery disease” OR “CAD” OR “coronary heart disease” OR “CHD” OR “myocardial infarction” OR “MI” OR “ischemic cardiovascular disease” OR “ischemic cardiovascular events” OR “stroke” OR “cerebrovascular disease” OR “ischemic cerebrovascular events” OR “cerebral infarction” OR “cerebral ischemia” OR “brain infarction” OR “carotid artery stenosis” OR “CAAD” OR “transient ischemic attack” OR “TIA” OR “peripheral Arterial Disease” OR “PAD” OR “peripheral artery occlusive disease” OR “PAOD” OR “renal artery stenosis” OR “RAS” OR “retinal artery occlusion” OR “RAO” OR “aortic aneurysm” OR “atherosclerosis”). In addition, all references mentioned in the identified original articles were reviewed by hand-searching in order to investigate additional literature that was not indexed. | Literature search |
| Study selection | 9 | The flow diagram of the study selection for this meta-analysis was shown in the Fig. 1. A total of 1003 articles were identified after searching. After careful review, 47 articles involving 58 studies (42 studies for R219K polymorphism and 16 studies for M883I polymorphism) met the inclusion criteria and were selected in this meta-analysis [2-48]. For the ABCA1 R219K polymorphism, 12551 AS cases and 19548 controls were included in this study to assess the association between this variant and AS risk [2-41]. For the ABCA1 M883I polymorphism, 4224 AS cases and 3462 controls were identified to assess the association of the variant with AS risk [14,15,22,23,29,30,42-48]. | Characteristics of the studies |
| Data collection process | 10 | Data were independently extracted from original publications by two reviewers (Yin YW and Zhang LL) according to the inclusion criteria listed above. Discrepancy between the reviewers was resolved by consensus or a third reviewer (Li JC). | Data extraction |
| Data items | 11 | Data, including name of the first author, year of publication, study population (country, ethnicity), type of atherosclerotic disease, source of controls (population-based studies and hospital-based studies), sample size (total numbers of cases and controls), and number of genotypes in cases and controls, were extracted from each study. | Data extraction |
| Risk of bias in individual studies | 12 | The quality of the individual studies was evaluated and scored by two reviewers independently based on the Newcastle-Ottawa Scale (NOS) [56]. Each study was assessed based on three broad perspectives: selection, comparability, and exposure), and each satisfactory answer received one point. The NOS ranges between zero (none of the quality criterion was met) up to nine stars (all the quality criteria were met), and the high-quality study was considered as the one with a score equal to or higher than seven. The third reviewer (Li BH) examined the results, and a consensus was reached. | Quality assessment for individual studies |
| Summary measures | 13 | The principal summary measures are odds ratios(ORs) and 95% confidence intervals (CIs). | Statistical analysis |
| Synthesis of results | 14 | The combined ORs were respectively calculated for allelic model (K allele vs. R allele for R219K; I allele vs. M allele for M883I), additive model (K/K vs. R/R for R219K; I/I vs. M/M for M883I), recessive model (K/K vs. R/K+R/R for R219K; I/I vs. M/I+M/M for M883I), and dominant model (K/K+R/K vs. R/R for R219K; I/I+M/I vs. M/M for M883I). Heterogeneity between studies was formally tested by using Cochran’s Q statistic and considered statistically signiﬁcant when p＜0.10. Heterogeneity was also measured with I^2^ statistic (I^2^>50% indicated evidence of heterogeneity). The fixed-effects model was used in the absence of between-study heterogeneity; otherwise the random-effects model was used. | Statistical analysis |

Page 1 of 2

| **Section/topic** | **#** | **Checklist item** | **Reported on page #** |
| --- | --- | --- | --- |
| Risk of bias across studies | 15 | An estimate of potential publication bias was carried out by Begg’s funnel plot and Egger’s regression test (p < 0.05 was considered representative of statistically signiﬁcant publication bias) | Statistical analysis |
| Additional analyses | 16 | Subgroup analyses were performed based on ethnicity (Europeans and Asians), atherosclerotic disease (CAD and IS) and source of controls (population-based studies and hospital-based studies). Sensitivity analyses were performed by limiting the meta-analysis to studies conforming to HWE and the high quality studies (according to the NOS score). Galbraith plot was used to detect the potential sources of heterogeneity. | Statistical analysis |
| **RESULTS** | | |  |
| Study selection | 17 | The flow diagram of the study selection for this meta-analysis was shown in the Fig. 1. A total of 1003 articles were identified after searching. After careful review, 47 articles involving 58 studies (42 studies for R219K polymorphism and 16 studies for M883I polymorphism) met the inclusion criteria and were selected in this meta-analysis [2-48]. For the ABCA1 R219K polymorphism, 12551 AS cases and 19548 controls were included in this study to assess the association between this variant and AS risk [2-41]. For the ABCA1 M883I polymorphism, 4224 AS cases and 3462 controls were identified to assess the association of the variant with AS risk [14,15,22,23,29,30,42-48]. | Characteristics of the studies |
| Study characteristics | 18 | Table 1 shows the studies included in the meta-analysis and their main characteristics. | Characteristics of the studies |
| Risk of bias within studies | 19 | The NOS results were shown in Table 1. The NOS results showed that the average scores were 6.9 and 6.8, respectively. | Characteristics of the studies |
| Results of individual studies | 20 | The main results of individual studies were shown in Fig 2 and Fig 3 (Fig. 2 and Fig. 3: Forest plots for the ABCA1 R219K and M883I polymorphisms and AS risk in different genetic models.). | Quantitative synthesis |
| Synthesis of results | 21 | For the ABCA1 R219K polymorphism, The overall results showed evidence of significant association between the ABCA1 R219K polymorphism and the susceptibility to AS, suggesting that the ABCA1 R219K polymorphism was a protective role for AS (for K allele vs. R allele: OR = 0.77, 95% CI = 0.71-0.84, p < 0.01; for K/K vs. R/R: OR = 0.60, 95% CI = 0.51-0.71, p < 0.01; for K/K vs. R/K+R/R: OR = 0.69, 95% CI = 0.60-0.80, p < 0.01; for K/K+R/K vs. R/R: OR = 0.74, 95% CI = 0.66-0.83, p < 0.01). For the ABCA1 M883I polymorphism, there was significant association between the ABCA1 M883I polymorphism and the susceptibility to AS in the allelic model (for I allele vs. M allele: OR = 0.85, 95% CI = 0.78-0.93, p < 0.01). However, no obvious evidence of association was found in the additive model (for I/I vs. M/M: OR = 0.79, 95% CI = 0.63-1.01, p = 0.06), recessive model (for I/I vs. M/I+M/M: OR = 0.92, 95% CI = 0.75-1.14, p = 0.45) and dominant model (for I/I+M/I vs. M/M: OR = 0.86, 95% CI = 0.72-1.02, p = 0.08). The main results of meta-analysis were shown in Fig. 2-3 and Table 2, respectively. | Quantitative synthesis |
| Risk of bias across studies | 22 | Begg’s funnel plot and Egger’s regression test were performed to assess potential publication bias. For the ABCA1 R219K polymorphism, visual inspection of the funnel plot (Fig.6: K allele vs. R allele) displays asymmetrical distribution of OR estimations, suggesting significant publication bias. In addition, the results of Egger’s regression test also provided evidence for publication bias ( p < 0.01 for allelic model, p < 0.01 for additive model, p < 0.01 for recessive model, and p < 0.01 for dominant model, respectively). For the ABCA1 M883I polymorphism, no obvious asymmetry was observed in any genetic model according to the visual assessment of funnel plot (Fig.6: I allele vs. M allele). Moreover, the results of Egger’s regression test did not provide any statistical evidence for publication bias (p = 0.34 for allelic model, p = 0.24 for additive model, p = 0.10 for recessive model, and p = 0.09 for dominant model, respectively). | Publication bias |
| Additional analysis | 23 | **Subgroup analyses:** For the ABCA1 R219K polymorphism, significant associations were also found between this variant and the susceptibility to AS in the Asians group (for K allele vs. R allele: OR = 0.71, 95% CI = 0.64-0.79, p < 0.01; for K/K vs. R/R: OR = 0.52, 95% CI = 0.42-0.63, p < 0.01; for K/K vs. R/K+R/R: OR = 0.62, 95% CI = 0.52-0.75, p < 0.01; for K/K+R/K vs. R/R: OR = 0.66, 95% CI = 0.59-0.74, p < 0.01), CAD group (for K allele vs. R allele: OR = 0.74, 95% CI = 0.67-0.82, p < 0.01; for K/K vs. R/R: OR = 0.56, 95% CI = 0.47-0.67, p < 0.01; for K/K vs. R/K+R/R: OR = 0.64, 95% CI = 0.55-0.74, p < 0.01; for K/K+R/K vs. R/R: OR = 0.72, 95% CI = 0.64-0.82, p < 0.01), IS group (for K/K+R/K vs. R/R: OR = 0.80, 95% CI = 0.68-0.94, p < 0.01), population-based group (for K allele vs. R allele: OR = 0.76, 95% CI = 0.67-0.85, p < 0.01; for K/K vs. R/R: OR = 0.58, 95% CI = 0.47-0.72, p < 0.01; for K/K vs. R/K+R/R: OR = 0.68, 95% CI = 0.56-0.82, p < 0.01; for K/K+R/K vs. R/R: OR = 0.72, 95% CI = 0.62-0.83, p < 0.01) and hospital-based group (for K allele vs. R allele: OR = 0.81, 95% CI = 0.71-0.92, p < 0.01; for K/K vs. R/R: OR = 0.65, 95% CI = 0.51-0.82, p < 0.01; for K/K vs. R/K+R/R: OR = 0.75, 95% CI = 0.645-0.87, p < 0.01; for K/K+R/K vs. R/R: OR = 0.80, 95% CI = 0.68-0.94, p < 0.01), respectively. For the ABCA1 M883I polymorphism, significant associations were found between this variant and the susceptibility to AS in the Asians group (for I allele vs. M allele: OR = 0.82, 95% CI = 0.74-0.91, p < 0.01; for I/I vs. M/M: OR = 0.72, 95% CI = 0.56-0.93, p = 0.01; for I/I+M/I vs. M/M: OR = 0.75, 95% CI = 0.61-0.91, p < 0.01), CAD group (for I allele vs. M allele: OR = 0.82, 95% CI = 0.74-0.90, p < 0.01; for I/I vs. M/M: OR = 0.73, 95% CI = 0.57-0.94, p = 0.01; for I/I+M/I vs. M/M: OR = 0.81, 95% CI = 0.68-0.97, p = 0.02), IS group (for I allele vs. M allele: OR = 1.43, 95% CI = 1.01-2.02, p = 0.04), and population-based group (for I allele vs. M allele: OR = 0.87, 95% CI = 0.79-0.96, p < 0.01), respectively. The main results of subgroup analyses were shown in Table 2.  **Sensitivity analyses:** Sensitivity analyses were conducted to determine whether modification of the inclusion criteria of the meta-analysis affected the final results. The included studies were limited to those conforming to HWE or the high quality studies (NOS score ≥7). Overall, the corresponding pooled ORs were not materially altered, either for the ABCA1 R219K polymorphism or for M883I polymorphism. This suggested that the overall results of this meta-analysis were statistically robust. The main results of sensitivity analyses were shown in Table 2.  **Heterogeneity analysis:** For the ABCA1 R219K polymorphism, signiﬁcant heterogeneity existed in the overall comparisons (for allelic model: *P_Q_* = 0.00, I^2^ = 77.4%; for additive model: *P_Q_* = 0.00, I^2^ = 67.9%; for recessive model: *P_Q_* = 0.00, I^2^ = 64.2%; for dominant model: *P_Q_* = 0.00, I^2^ = 69.7%). For the ABCA1 M883I polymorphism, signiﬁcant heterogeneity existed in the dominant model (for *P_Q_* = 0.00, I^2^ = 64.3%). In contrast, the allelic model, additive model and recessive model did not present significant heterogeneity (for allelic model: *P_Q_* = 0.24, I^2^ = 19.5%; additive model: *P_Q_* = 0.37, I^2^ = 7.9%; for recessive model: *P_Q_* = 0.75, I^2^ = 0 %). To clarify the sources of heterogeneity, we conducted the subgroup and sensitivity analyses. For the ABCA1 R219K polymorphism, we did not effectively remove the heterogeneity in the overall comparisons. For the ABCA1 M883I polymorphism, we only removed the heterogeneity in the Europeans group, IS group and HWE-based group. In addition to the above analyses, we also created a Galbraith plot to graphically assess the sources of heterogeneity. A total of 16 studies [3,12-14,16,17,21,24,27-30,33,35,37,39] were identified as the main contributors to heterogeneity in the four models of the ABCA1 R219K polymorphism (Fig. 4), and three studies [29,42,46] were identified as the main contributors to heterogeneity in the dominant model of the ABCA1 M883I polymorphism (Fig. 5). After excluding the outlier studies, the heterogeneity was effectively removed, respectively (data not shown). | Quantitative synthesis, Sensitivity analysis, and Heterogeneity analysis |
| **DISCUSSION** | | |  |
| Summary of evidence | 24 | The findings showed that the ABCA1 R219K and M883I polymorphisms may exert an reduced risk effect on AS. For the ABCA1 R219K polymorphism, the risk of developing AS in K allele carriers was 1.30-fold higher than those without. Furthermore, the individuals with K/K genotype had a significantly lower risk for developing AS (for OR=0.60 in additive model and OR=0.69 in recessive model) compared to those with R/K genotype and R/R genotype. As for the ABCA1 M883I polymorphism, the combined evidence showed that the individuals with I allele had a significantly lower risk for developing AS (for OR=0.85 in allelic model) compared to those without. Therefore, it is reasonable to assume that the ABCA1 R219K K allele and M883I I allele are the protective factors for the development of AS. | Discussion |
| Limitations | 25 | **Limitations:** Firstly, there was significant between-study heterogeneity in this meta-analysis. Heterogeneity may affect the precision of overall results, despite the use of appropriate meta-analytic techniques with random-effects model. Secondly, in the subgroup analyses, the sample sizes in some subgroup, such as the IS group and the hospital-based group of the ABCA1 M883I polymorphism, were relatively small, not having enough statistical power to explore the real association. Thirdly, publication bias was a potential problem that may bias the present results. Due to language restriction, some inevitable publication bias may exist in the present meta-analysis. Moreover, AS is a complex disease, involving potential interactions among gene-gene and gene-environment. A single gene polymorphism is insufﬁcient to provide the complete explanation of genetic risk for AS. However, many eligible studies included in this meta-analysis didn’t consider the environmental factors. | Discussion |
| Conclusions | 26 | **Conclusion：**In conclusion, the present meta-analysis suggests that the ABCA1 R219K and M883I polymorphisms are associated with the susceptibility to AS, especially in Asians. However, the result should be interpreted with caution because of its limitations. Further studies with large sample size, especially with the consideration of gene-gene and gene-environment interactions, will be needed to confirm our findings. | Discussion |
| **FUNDING** | | |  |
| Funding | 27 | This work was supported by Natural Science Foundation Project of CQ CSTC (CSTC2012JJJQ10003 to Li-li Zhang) and National Natural Science Foundation of China ( NSFC 81271282 to Jing-cheng Li) | Online submission system |

*From:*  Moher D, Liberati A, Tetzlaff J, Altman DG, The PRISMA Group (2009). Preferred Reporting Items for Systematic Reviews and Meta-Analyses: The PRISMA Statement. PLoS Med 6(6): e1000097. doi:10.1371/journal.pmed1000097

For more information, visit: **www.prisma-statement.org**.

Page 2 of 2
